# Multivariate longitudinal data for survival analysis of cardiovascular event prediction in young adults: insights from a comparative explainable study

**DOI:** 10.1186/s12874-023-01845-4

**Published:** 2023-01-25

**Authors:** Hieu T. Nguyen, Henrique D. Vasconcellos, Kimberley Keck, Jared P. Reis, Cora E. Lewis, Steven Sidney, Donald M. Lloyd-Jones, Pamela J. Schreiner, Eliseo Guallar, Colin O. Wu, João A.C. Lima, Bharath Ambale-Venkatesh

**Affiliations:** 1 Department of Biomedical Engineering, Johns Hopkins University, Baltimore, MD USA; 2 Department of Cardiology, Johns Hopkins University, Baltimore, MD USA; 3 National Heart, Lung, and Blood Institute, Bethesda, MD USA; 4 Department of Epidemiology, School of Public Health, University of Alabama at Birmingham, Birmingham, AL USA; 5 Division of Research, Kaiser Permanente, Oakland, CA USA; 6 Department of Preventive Medicine, Northwestern University, Chicago, IL USA; 7 School of Public Health, University of Minnesota, Minneapolis, MN USA; 8 Department of Epidemiology, Johns Hopkins University School of Public Health, Baltimore, MD USA; 9 Department of Radiology, Johns Hopkins University, Baltimore, MD USA

**Keywords:** Longitudinal data, Explainable AI, Survival analysis, Risk prediction, Repeated measures, Personalized medicine, Time-varying covariates, SHAP, TIME, CARDIA

## Abstract

**Background:**

Multivariate longitudinal data are under-utilized for survival analysis compared to cross-sectional data (CS - data collected once across cohort). Particularly in cardiovascular risk prediction, despite available methods of longitudinal data analysis, the value of longitudinal information has not been established in terms of improved predictive accuracy and clinical applicability.

**Methods:**

We investigated the value of longitudinal data over and above the use of cross-sectional data via 6 distinct modeling strategies from statistics, machine learning, and deep learning that incorporate repeated measures for survival analysis of the time-to-cardiovascular event in the Coronary Artery Risk Development in Young Adults (CARDIA) cohort. We then examined and compared the use of model-specific interpretability methods (Random Survival Forest Variable Importance) and model-agnostic methods (SHapley Additive exPlanation (SHAP) and Temporal Importance Model Explanation (TIME)) in cardiovascular risk prediction using the top-performing models.

**Results:**

In a cohort of 3539 participants, longitudinal information from 35 variables that were repeatedly collected in 6 exam visits over 15 years improved subsequent long-term (17 years after) risk prediction by up to 8.3% in C-index compared to using baseline data (0.78 vs. 0.72), and up to approximately 4% compared to using the last observed CS data (0.75). Time-varying AUC was also higher in models using longitudinal data (0.86–0.87 at 5 years, 0.79–0.81 at 10 years) than using baseline or last observed CS data (0.80–0.86 at 5 years, 0.73–0.77 at 10 years). Comparative model interpretability analysis revealed the impact of longitudinal variables on model prediction on both the individual and global scales among different modeling strategies, as well as identifying the best time windows and best timing within that window for event prediction. The best strategy to incorporate longitudinal data for accuracy was time series massive feature extraction, and the easiest interpretable strategy was trajectory clustering.

**Conclusion:**

Our analysis demonstrates the added value of longitudinal data in predictive accuracy and epidemiological utility in cardiovascular risk survival analysis in young adults via a unified, scalable framework that compares model performance and explainability. The framework can be extended to a larger number of variables and other longitudinal modeling methods.

**Trial registration:**

ClinicalTrials.gov Identifier: NCT00005130, Registration Date: 26/05/2000.

**Supplementary Information:**

The online version contains supplementary material available at 10.1186/s12874-023-01845-4.

## Background

The rapidly expanding availability of large health data sets and recent advances in computing power have fuelled the growing literature on risk prediction models for health outcomes. Prediction of risk for an adverse event holds much potential for preventive, interventional, and monitoring strategies as well as shedding light on natural history and pathophysiology. Even though most large datasets contain repeated measurements of same variable at different time points (longitudinal data), only a small fraction of prediction models have routinely incorporated longitudinal data. Less than 8% of prediction models in studies published from 2009 to 2016 included longitudinal data as time-varying covariates, while many studies only rely on data at a single time point (cross-sectional data), typically at baseline, potentially discarding valuable information from the longitudinal dataset [1]. The utility of longitudinal data for long-term risk prediction is mixed in the literature. For example, in cardiovascular disease (CVD) risk prediction, several studies have reported that longitudinal data improve prediction [2–4], while some observed negligible to no difference compared to using only baseline data [5–7]. However, most studies only consider a small number (8 at most) of time-varying covariates and mostly focus on traditional risk factors such as blood pressure and total cholesterol. The utility of higher-dimensional longitudinal data for CVD risk prediction remains poorly understood, especially in the young adult population, for whom accurate risk stratification at a younger age could potentially have a great impact on an individual’s life course [8]. Investigations are needed in this area to determine the value of higher-dimensional longitudinal data.

Several approaches have been proposed for dealing with longitudinal data. One of the most common entails the inclusion of summary statistics in the prediction model, such as average over time, linear slope, or cumulative exposure (area under the time-exposure curve). However, these approaches may not fully capture the rich information contained in longitudinal datasets such as variability and timing of exposure, from longitudinal data. Another increasingly popular strategy is joint modeling (JM), which is in essence simultaneously fitting and combining the longitudinal and survival processes [9, 10]. Reviews of JM methods have been described elsewhere [9, 10]. However, papers using JM in CVD have been limited to a few longitudinal variables at most [11], and the capability of JM in handling higher-dimensional data remains unclear. Machine Learning (ML) methods have been the solution for higher-dimensional data. Numerous works have demonstrated the use of ML in risk prediction of general and CVD medical outcomes in particular [12–16]. However, the majority of these studies employ ML classification-based methods and not survival analysis and thus are limited in several aspects when dealing with time-to-event outcomes. For example, ML classifiers cannot predict the time to event, do not account for censoring, need to be re-trained for each prediction time, and could have inconsistent predictions at different times (e.g., classifying a patient as having CVD at month 5 but CVD-free at month 10) [17, 18]. Among frequently used ML methods designed for survival analysis such as Random Survival Forest (RSF) [19], DeepSurv [20], and Nnet-survival [21], many cannot directly process the time series of repeated measures as input. A couple of prototyped ML survival algorithms for time series have been introduced recently, such as Dynamic-DeepHit and MATCH-Net [22, 23], but their utilities need to be externally validated in medical applications. Among all the different strategies to incorporate repeated measures, it is unclear which strategy would be the most useful in the analysis of large datasets obtained across a long period of time.

When assessing usefulness, the quantification of predictive superiority is often performed using performance metrics such as AUC or C-index, which may be insufficient in determining clinical utility [24]. Model interpretability and clinical implications are infrequently taken into consideration, despite being the main reasons limiting adoption of longitudinal data in prediction modelling [25]. Temporal dependencies among repeated measures challenge longitudinal data interpretability [26], and even when interpretability is feasible, clinical utility may not be assured. Indeed, model interpretability and clinical value remain pivotal questions in the assessment of longitudinal data based predictive models.

This study has several objectives to tackle the challenges described above. We first aim to evaluate the utility of multivariate longitudinal data for survival analysis of incident CVD prediction in young adults. For this purpose, we apply 6 distinct strategies from statistics, statistical ML, and deep learning to process longitudinal data and predict the time-to-cardiovascular event in the Coronary Artery Risk Development in Young Adults (CARDIA) population. Secondly, we compare the predictive value among those strategies and against models using only cross-sectional data (data collected once, such as in the baseline or most recent exam). Finally, we apply ML-based model-specific and model-agnostic explainability methods to explain the top-performing models and derive appropriate clinical insights.

## Methods

In this section, we present the dataset and cohort filtering in the first subsection, then we present our modeling and analysis framework in 3 subsequent subsections. The analysis was performed in R and Python.

### CARDIA, study design, participant selection, and outcome definition

In brief, our study used longitudinal data from the first six follow-up exam visits of a larger study called CARDIA for survival analysis of future CV events. The design of the CARDIA study has been described previously [27]. In brief, CARDIA is a prospective, population-based observational cohort study of 5114 (originally 5115, one person withdrew consent) White and Black men and women aged 18 to 30 years, at enrollment in 1985-86. Study participants were recruited at four centers in the United States (Birmingham, AL.; Chicago, IL; Minneapolis, MN; and Oakland, CA). The cohort is approximately balanced in age, race, sex, and educational level.

**Fig. 1:**
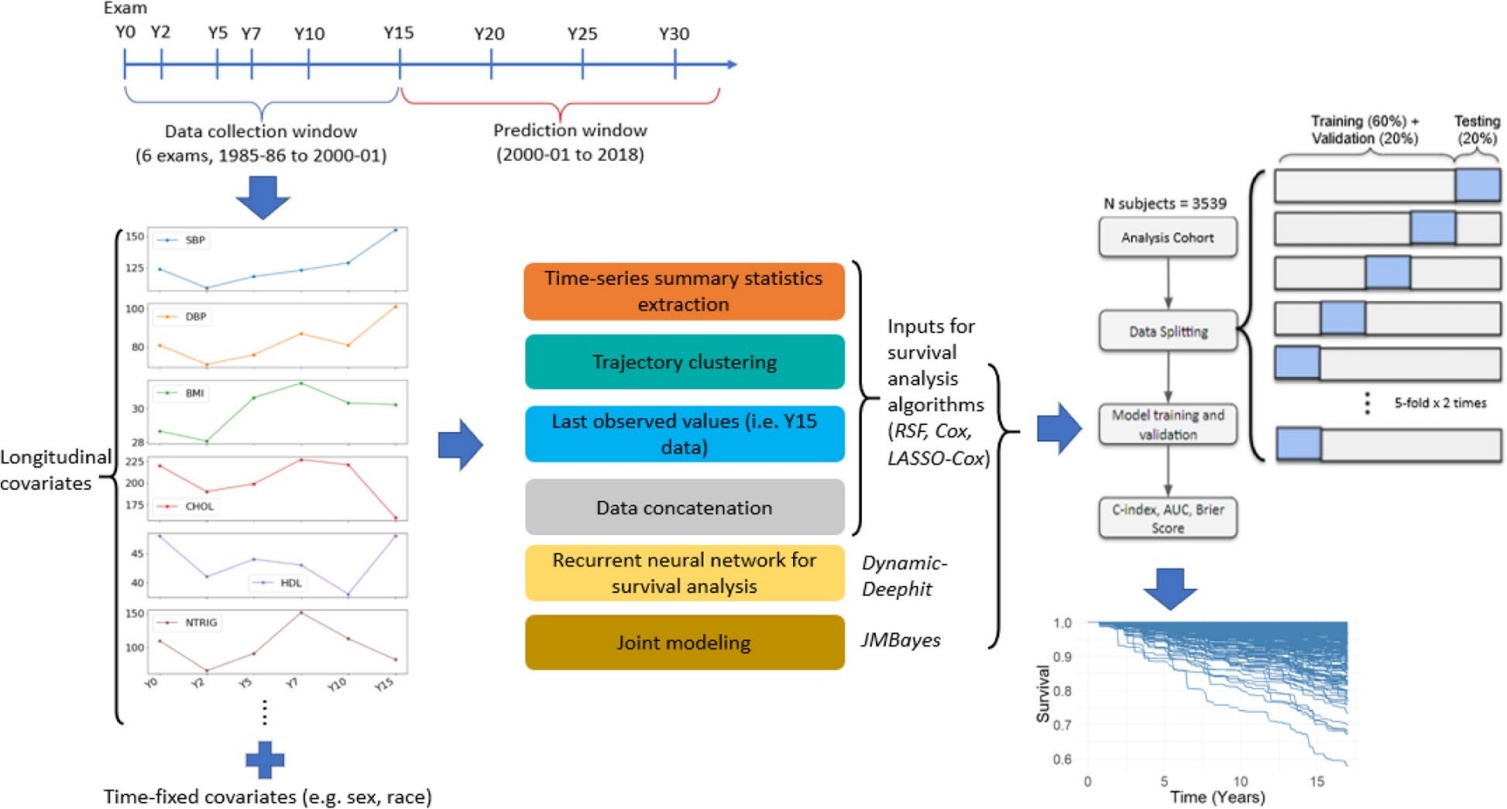
Method framework visualization. Data from the first six exam visits were used for prediction of time-to-CVD event in the subsequent 17 years. Six strategies were employed to incorporate longitudinal data in 35 variables. All models were trained and tested under the same 5-fold x 2 times cross-validation scheme. Model output was survival probabilities (1-predicted CVD risk) over time. Model performance was quantified by C-index, AUC, Brier Score, and other metrics. CVD: cardiovascular disease; Cox: Cox proportional hazards; LASSO-Cox: Cox Proportional Hazards penalized by Least Shrinkage and Selection Operator; Dynamic-Deephit: recurrent neural network-based survival method for longitudinal data; JMBayes: joint modeling under Bayesian approach; RSF: random survival forest

To investigate the utility of longitudinal data for long-term CVD risk prediction, we used data from six exams (Year 0 (Y0): 1985-86, Y2: 1987-88, Y5: 1990-91, Y7: 1992-93, and Y15: 2000-01), also known as the data collection window (Fig. 1). Each exam collected a wide variety of variables believed to be related to heart disease. The reasons Y15 was chosen as the last exam for data collection were that the CVD rate was relatively flat from Y0 to Y15 (very few events) and increased linearly from Y15 onwards (Fig. S1), and that Y15 still allowed us to capture premature CVD events. The prediction window started from after Y15 through August 2018, with the endpoint being the first CVD event during these 17 years, death or loss to follow-up. An incident CVD event included coronary heart disease (CHD – myocardial infarction, acute coronary syndrome, or CHD death, including fatal myocardial infarction), stroke, transient ischemic attack (TIA), hospitalization for heart failure, intervention for peripheral arterial disease, or death from cardiovascular causes. The outcome ascertainment protocols have been described in detail elsewhere [28, 29] and are included in the Supplement. The final cohort consisted of 3539 participants with 19,988 total visits. The participants were required to have all six exams. The full inclusion/exclusion criteria for the final cohort are shown in the cohort selection flowchart in Fig. S2.

We included a total of 35 variables that were repeatedly measured in 6 exams from Y0 to Y15 in most participants. Besides demographic variables such as race, age, and sex being the time-fixed variables, the rest were considered longitudinal and included information about anthropometry (BMI, weight, waist girth), physiological measures (blood pressure, pulse), indication of taking anti-hypertensive medication, socioeconomic markers (education, ability to provide for the basics), medical history, alcohol use, smoking, lipids (cholesterol, triglycerides), glucose, marijuana use, and physical activity levels. A full list of variables describing their coding and abbreviation is shown in Table S1 and their distributions are shown in Table 1. Most variables had no missing data in all participants, and in the rare cases of missing data in a certain exam, last observation carried forward was used. A total of 88 participants out of 3539 (< 3%) were excluded due to nonavailability of data. Considering the small amount of missing data, we only considered data with no missingness in this study. In this proof-of-concept study, we wanted to compare strategies using the same set of data and minimize external pre-processing steps that could confound or introduce bias to the data, so we aimed to evaluate them on the same set of data (without missingness). In summary, we used 32 longitudinal variables in a total of 35 variables collected in six exams spanning 15 years for the survival analysis of future CVD in the subsequent 17 years.

**Table 1:** Characteristics of CARDIA participants at baseline (Y0, 1985–1986) and at Y15 Exam (2000–2001). Values outside parentheses denote mean, values inside parentheses denote standard deviation unless noted to be in percentage

Variable	Y0 (all participants)	Y0	Y15	Variable	Y0 (all participants)	Y0	Exam Y15
	(*N* = 5114^a^)	(*N* = 3539)	(*N* = 3539)		(*N* = 5114^a^)	(*N* = 3539)	(*N* = 3539)
Age	25 (3.6)	25 (3.6)	40 (3.6)				
Sex (Male)	2327 (46%)	1572 (44%)	1572 (44%)	# of times pregnant in life	0.60 (1.2)	0.59 (1.2)	1.4 (1.9)
Race				Asthma	219 (4%)	141 (4%)	204 (6%)
Black	2637 (52%)	1666 (47%)	1666 (47%)	Cancer	141 (3%)	106 (3%)	115 (3%)
White	2477 (48%)	1873 (53%)	1873 (53%)	Diabetes	43 (1%)	22 (1%)	198 (6%)
Systolic blood pressure (SBP)	110 (11)	110 (11)	113 (15)	Gall bladder problem	45 (1%)	31 (1%)	114 (3%)
Diastolic blood pressure (DBP)	69 (9.6)	69 (9.5)	74 (11)	Kidney problem	215 (4%)	130 (4%)	216 (6%)
Use of anti-hypertensive medication	115 (2%)	75 (2%)	253 (7%)	Nervous, emotional or mental disorder	380 (7%)	253 (7%)	227 (6%)
Pulse beats (in 30s)	35 (5.5)	34 (5.3)	34 (5.7)	Liver problem	54 (1%)	39 (1%)	48 (1%)
Body Mass Index (BMI)	24 (5.0)	24 (4.9)	29 (6.8)	Parent history of heart attack	733 (14%)	514 (15%)	718 (20%)
Waist girth (cm)	78 (11)	78 (11)	89 (15)	Smoking now	1546 (30%)	947 (27%)	772 (22%)
Weight (lbs.)	157 (36)	157 (35)	185 (47)	Cigarettes smoked/day	5.5 (9.0)	5.1 (8.7)	2.7 (6.7)
Arm circumference (cm)	29 (4.7)	29 (4.7)	33 (5.3)	# of drinks of beer/week (12 oz/drink)	3.0 (6.9)	3.0 (6.9)	2.4 (7.3)
Education level (grades)	14 (2.3)	14 (2.2)	15 (2.9)	# of shots of hard liquor/week	1.0 (3.0)	0.96 (2.9)	0.94 (3.6)
Ability to pay for the very basics				# of drinks of wine/week (5 oz/drink)	0.85 (2.2)	0.85 (2.2)	1.2 (3.6)
Very hard	221 (4%)	126 (4%)	113 (3%)	Times used marijuana in life	2.0 (1.5)	2.1 (1.5)	2.2 (1.8)
Hard	357 (7%)	240 (7%)	166 (5%)	Physical activity level (self-assessed answers)			
Somewhat hard	1212 (24%)	827 (23%)	726 (21%)	1: Inactive	367 (7%)	241 (7%)	228 (6%)
Not very hard	3315 (65%)	2340 (66%)	2534 (72%)	2	732 (14%)	508 (14%)	593 (17%)
Total cholesterol (mg/dL)	177 (33)	177 (33)	185 (36)	3: Moderately active	1965 (38%)	1363 (39%)	1570 (44%)
Use of cholesterol-lowering medication	11 (0.2%)	9 (0.3%)	9 (0.3%)	4	1088 (21%)	795 (22%)	617 (17%)
HDL cholesterol (mg/dl)	53 (13)	53 (13)	51 (14)	5: very active	955 (19%)	629 (18%)	531 (15%)
LDL cholesterol (mg/dl)	110 (31)	110 (31)	113 (32)	Outcomes:			
Triglycerides (mg/dl)	73 (48)	73 (47)	105 (92)	All-cause death by end of follow-up	468 (9%)	200 (6%)	200 (6%)
Fasting glucose (mg/100 ml)	83 (16)	82 (10)	89 (16)	CVD event by end of follow up	287 (6%)	181 (5%)	181 (5%)

^a^1 participant withdrew from the original enrollment cohort of 5115

### Modeling longitudinal data

In this work, we examined six different strategies to analyze longitudinal data. The first four strategies were essentially two-step procedures, in which the first step was a processing step that transformed the longitudinal trajectory input into a tabular format (i.e. each input feature held a single value instead of a sequence of repeated measures), then the second step fed the inputs from the first step to a survival method (e.g., RSF). The last two modeling strategies directly incorporated raw longitudinal data for survival prediction as the input (Fig. 1). The six strategies were as below:

1. Time series massive summary statistics extraction.
2. Trajectory clustering.
3. Last observed values.
4. Data concatenation.
5. Recurrent neural network for survival analysis.
6. Joint modeling.

The first strategy derived summary statistics from the trajectories. For each trajectory corresponding to each variable, we extracted not only the commonly used statistics such as minimum, maximum, mean, variance, linear trend intercept, and slope, but also many lesser-known statistics such as time-reversal asymmetry statistic, autocorrelation, c3 statistic, absolute energy, entropy measures, change in quantiles, different correlation measures, results from time series hypothesis tests, coefficients from time series transformations, etc. The approach has also been called highly comparative time series analysis [30]. We hypothesized that the lesser-known statistics could capture more fine-grained information than just using simple statistics and that the combination of many kinds of time series statistics would fully capture the longitudinal and cross-sectional trends and variations in the data. We used the Python package *tsfresh* [31] for efficient and automated extraction of hundreds of features for each trajectory. The features were then pruned by removing features with at least one NA value, features with only one unique value, and correlated features with a threshold of 0.95 spearman correlation. The pruned features along with the three demographic variables (age, sex, and race) were then fed as inputs to survival analysis algorithms such as RSF, Cox, and Cox penalized by LASSO (least absolute shrinkage and selection operator) (LASSO-Cox) to predict CVD events.

The second strategy essentially performed clustering of the trajectories of each longitudinal variable, then used the assigned cluster membership per variable per participant as the inputs for survival algorithms. Trajectory clustering has been used previously in CARDIA [4, 32, 33], however, its utility as input for ML survival analysis models is underexplored. We used a 3-step trajectory clustering approach using the R package *traj* package [34, 35], which resembled the *Proc-Traj* in SAS [36]. The first step calculated 24 summary measures that described features of the trajectories. The second step performed a factor analysis on these 24 measures to select measures that best describe the main features of the trajectories and removed redundant measures. The third step assigned the trajectories into clusters by applying the k-means algorithm to the previously selected measures. The optimal number of clusters for k-means was determined algorithmically by experimenting k-means with 23 clustering criteria from the R package *NbClust* [37], including many widely used criteria to lesser-known ones, including the within-cluster sum of squares (the ‘elbow’ method), gap statistic, silhouette score, Calinski and Harabasz index, cubic clustering criterion, and information criterion [37]. The predictive comparison of different criteria is shown in Fig. S3 in the Supplement.

The third modeling strategy was simply using the last observed value from the longitudinal data as input for survival algorithms like RSF and LASSO-Cox. This approach could be thought of as a single landmark analysis. This method has been among the most common use of longitudinal data, for example using the last observed values in electronic health records to predict short-term outcomes [5, 24, 38]. Here, the last values up to the last time point of the data collection window, Y15, were used. In our study setting, the use of Cox on Y15 values to predict post-Y15 CVD is the equivalent to the extended Cox model for longitudinal exposures.

The fourth strategy, data concatenation, included all measurements across all different time points as separate predictors, by concatenating the repeated measurements of 32 longitudinal variables from six exams into a matrix of (32 × 6) variable columns. This matrix of 192 variables plus the time-fixed demographic variables was then fed as input to RSF and LASSO-Cox. This strategy, of course, strongly assumed that the data collected 5 years apart from different exams were independent of one another. However, this strategy has shown good predictive performance, for example including all past data points in electronic health records that were collected only a few days apart for 10-year CVD prediction [13, 39, 40]. Therefore, despite this strategy’s limitation of the independence assumption, we were interested in the predictive value of this strategy.

The fifth strategy captured the longitudinal history by embedding the history of past measures into latent representations to predict survival risks via deep learning. We adapted a recently introduced recurrent network-based method capable of incorporating longitudinal data for survival analysis called Dynamic-DeepHit [22]. This method issued dynamically updated survival predictions without making any assumptions about the underlying longitudinal and survival processes. Briefly, Dynamic-DeepHit is a multi-task network that consists of two sets of subnetworks, one is a shared subnetwork that handles longitudinal measurements and predicts the next measurements of time-varying covariates. The other set of subnetwork includes cause-specific survival networks which estimate the joint distribution of the first hitting time and event [41]. To compare the performance gain of this strategy in CVD prediction with other longitudinal-modeling strategies in this work, we applied this method to the same cohort and same longitudinal data, meaning participants with CVD or censorship during the data collection were removed and data outside the data collection window were not included. A separate model focusing on dynamic prediction using Dynamic-DeepHit that included all participants since Y0 was also developed and its performance in time-varying AUC is shown in Fig. S4 the Supplement.

The sixth and last strategy to incorporate longitudinal data was statistical joint modeling (JM), by simultaneously describing both longitudinal and survival processes. We implemented the JM method proposed by Rizopoulos that fit joint models using a Bayesian approach via the R package *JMbayes* [42]. The joint model consisted of two sets of sub-models: one set included 32 linear mixed models to model the 32 longitudinal variables, and the other set included a Cox model including the time-fixed variables to model the time-to-event process. These two sets of sub-models were linked via a function of shared random effects, and the joint model aimed to learn a full representation of the joint distribution of the longitudinal time-to-event data.

The six modeling strategies described above were compared with a reference strategy, which used only data from the first exam (Y0) as input to RSF, Cox, and LASSO-Cox. In some previous work, this strategy was referred to as the baseline carried forward method (BCF) [5].

### Model training and evaluation

All the models were trained and evaluated on the same cohort by 5-fold x 2 times cross-validation scheme (Fig. 1). For each time the whole data was split, 20% of the data went into testing, while the remaining 80% was further divided into training and validation sets. The training sets were used to fit the models, the validation sets were for hyperparameter tuning, and the testing sets were for assessing model performance. Stratified sampling was conducted to ensure the same ratio of events to non-events across the splits.

Model performance was quantified using several metrics. The main metrics include the time-dependent area under the receiver-operating curve (AUC) accounting for censorship [43], the time-dependent concordance index that accounted for censoring distribution [44], and the Brier Score [45]. We did not use Harrell’s C-index because Harrell’s version ignored the censoring distribution and assumed that the censoring distribution is independent of predictor variables [46]. The Brier Score measured the mean squared difference between the predicted probabilities and the actual outcomes. Higher C-index, higher AUC, and lower Brier Score indicated better prediction performance. In addition, the integrated AUC (iAUC) was used to quantify all time-varying AUCs as one number [47]. This iAUC was formulated for survival analysis and weighted by the estimated probability density of the time-to-event outcome [47]. We chose the AUC and iAUC as the main model performance metric because it had been shown that AUC was better than the C-index for the evaluation of t-year predicted risks [48]. Additional model performance metrics included sensitivity, specificity, positive predictive value (PPV), negative predictive value (NPP), F1 score, and Matthew’s Correlation coefficient (MCC) [49], at the last time point of the prediction window (17 years after Y15). We used the versions that had been adapted for survival analysis and accounted for censorship for these metrics [50, 51]. Sensitivity, Specificity, PPV, NPV, and MCC were determined at the probability binary cutoff where the F1 was maximized.

### Model interpretability

To explain the top-performing models, we employed model explanation methods that we believed to be the most appropriate to each top model and if possible, used the same explanation method among different models. For models representing modeling strategies that transformed longitudinal data to tabular formats (time series massive feature extraction, trajectory clustering, last observed value, and data concatenation), model-specific explanation methods for tabular data were suitable. We employed RSF’s permutation-based variable importance via the R package *rfsrc* [52] and the model-agnostic method SHapley Additive exPlanation (SHAP) via the Python package *shap* [53]. SHAP is an increasingly common explanation method that is based on game theory that essentially computes the contribution of a feature value to the difference between the actual prediction and the mean prediction. The model-agnostic version of SHAP, Kernel SHAP, was used [53]. We used the SHAP values from Kernel SHAP to obtain visualizations of how values of the top predictive features affected model predictions among all participants and in an individual participant.

RSF-VIMP and SHAP were not applicable for all models with input in a longitudinal format, such as models representing the deep learning and the joint modeling strategies, as they would ignore the temporal dependencies and correlations within the trajectory. An explanation method specifically designed for temporal data was therefore needed and a model-agnostic method was preferred to enable comparisons among models. Here we also adapted a third explanation method, Temporal Importance Model Explanation (TIME) [54]. TIME is a recent permutation-based model-agnostic method that can show, for each variable, its overall importance to the model, the most important temporal window, and whether the ordering of the values within the time window affects the model’s predictions [54]. We briefly explain TIME in the Supplement and the method is fully explained elsewhere [54]. We found that TIME was very computationally expensive and hence only used TIME to explain the best-performing model. It is worth noting that TIME is capable of explaining all models. TIME’s number of permutation parameter was set to 100, and the window localization parameter was set to 0.1, meaning the window of importance accounted for at least 90% of the total importance of the whole series for each variable.

## Results

A total of 3539 participants were included in the analysis. Table 1 describes the final analysis sub-cohort and the cohort of all CARDIA participants. Similar characteristics profiles were observed for the analysis sub-cohort and the all-participant cohort, except that there was a small decrease in the percentage of Black, male and smoking participants in the final analysis sub-cohort (2–3%). The mean age was 25 years old at CARDIA ExamY0 (baseline) and 40 at the start of the prediction window, Y15 (year 15 Exam). The final cohort consisted of 44% male, 47% black, and 53% white. Over 15 years within the data collection window, the averaged SBP and DBP (systolic and diastolic blood pressure), anthropometric measurements, total cholesterol, LDL cholesterol, triglycerides, and purchase ability (economic buying power) increased, while measures of smoking intensity reduced. At the end of the data collection window, 6% of the participants had diabetes, 6% had a history of kidney disease, 7% were taking anti-hypertensive medication, and 22% were current smokers. By the end of the prediction window, 181 CVD events had occurred (5%).

### Model performances

**Fig. 2:**
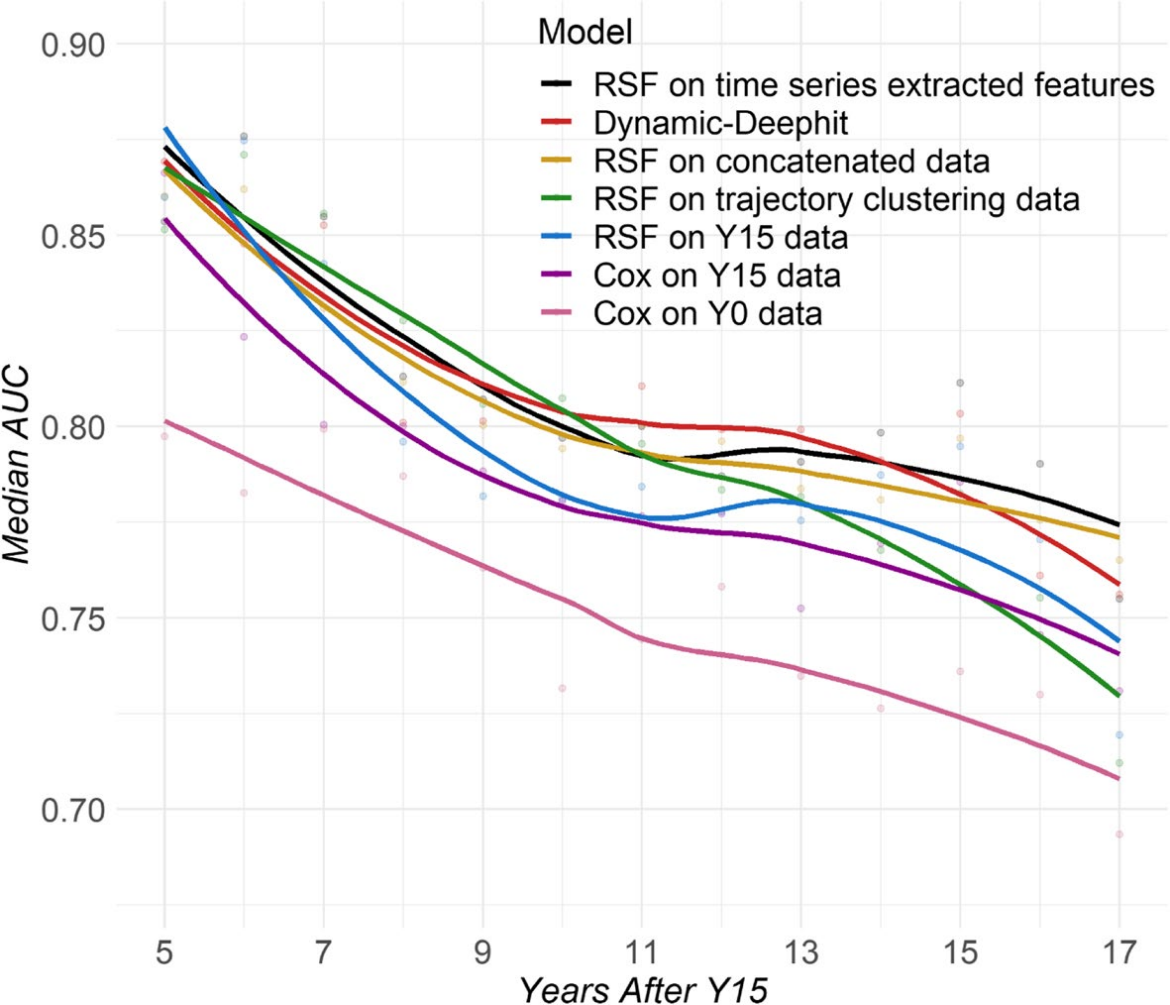
Model performance over time from different longitudinal modeling strategies. Median time-varying AUC over 10 test sets is shown for all six strategies (the joint model did not converge) plus the reference using only baseline (Y0) data. RSF: Random Survival Forest

Figure 2 shows the performances of representative models for each longitudinal modelling strategy over time. Table 2 shows the performances of all the prediction models using the main evaluation metrics (iAUC, C-index, and last AUC). The performances in additional evaluation metrics are provided in Table S2. Models using longitudinal data typically performed better than models using only Y15 data or Y0 data, in terms of AUCs and C-index. Models trained on Y0 data performed the worst, with 0.03–0.05 AUC lower compared to Y15-data training models and 0.05–0.07 compared to models trained on longitudinal data. The models using longitudinal data in at least two time-points and models using only Y15 data had similar performances for CVD prediction early in the prediction window, but the gap gradually widened to up to 0.03 in time-varying AUC with longer-term follow-up. Even though results from models using only Y15 data were only 0.016 lower in iAUC than models using longitudinal data, the Y15-only models were ~ 0.03 lower in post-10 years iAUC, C-index, and AUC at 17 years of follow-up. The models using longitudinal data had higher F1, PPV, and MCC than the cross-sectional models, relative differences were small across all models due to the low number of events in this younger population.

**Table 2:** Predictive performance of all models on 35 variables (mean and 95% empirical bootstrap interval)

Strategy	Model	iAUC	C-index	Last AUC
Time-series (TS) massive feature extraction	RSF on TS-extracted features	**0.808 (0.790, 0.826)**	**0.778 (0.757, 0.801)**	0.758 (0.733, 0.784)
	LASSO-Cox on TS-extracted features	0.744 (0.711, 0.781)	0.713 (0.686, 0.739)	0.701 (0.674, 0.727)
Recurrent neural network	Dynamic-DeepHit	0.794 (0.764, 0.825)	0.767 (0.745, 0.789)	**0.762 (0.733, 0.792)**
Trajectory clustering	RSF on trajectory clustering data	0.793 (0.772, 0.816)	0.741 (0.721, 0.76)	0.725 (0.705, 0.744)
Data concatenation	RSF on concatenated data	0.797 (0.778, 0.817)	0.766 (0.745, 0.788)	0.751 (0.725, 0.779)
Joint modeling	JMBayes	Did not converge		
Last observed values	RSF on Y15 data	0.793 (0.773, 0.812)	0.750 (0.729, 0.77)	0.731 (0.705, 0.76)
	Cox on Y15 data	0.778 (0.758, 0.804)	0.75 (0.733, 0.769)	0.728 (0.705, 0.752)
	Cox on Y15 data	0.793 (0.772, 0.818)	0.748 (0.73, 0.763)	0.727 (0.707, 0.745)
Reference (Y0 data)	RSF on Y0 data	0.754 (0.73, 0.777)	0.721 (0.698, 0.743)	0.699 (0.672, 0.726)
	Cox on Y0 data	0.748 (0.724, 0.773)	0.709 (0.686, 0.73)	0.685 (0.654, 0.716)
	LASSO-Cox on Y0 data	0.739 (0.713, 0.768)	0.698 (0.678, 0.717)	0.678 (0.645, 0.711)

The best scores are bolded. iAUC: integrated AUC, LASSO-Cox: Cox Proportional Hazards penalized by LeAst Shrinkage and Selection Operator. *JMBayes *Joint modeling with Bayesian approach, *RSF* Random Survival Forest

Among the models trained on longitudinal data, the RSF trained on time series extracted features (referred to as RSF-TS from now on) was the best performing model, with the highest iAUC, C-index, last AUC, F1, MCC, and smallest Brier Score. This model included a total of 249 features from 32 longitudinal variables. The model representing the deep learning approach, Dynamic-DeepHit, was comparable, with only a small dip in performance. The concatenation strategy model predicted CVD quite well, on par with Dynamic-DeepHit in the overall iAUC and the last C-index, despite ignoring time series self-correlation. The model for the trajectory clustering approach had the highest median time-varying AUC up to 10 years, however its performance dropped towards the prediction window endpoint. The joint modelling JMBayes model did not converge due to the algorithm being unable to handle the large number (32) of longitudinal variables. However, when the dimension was lower, for example, when the variable pool was limited to 9 traditional ASCVD risk factors (age, gender, race, SBP, cholesterol, HDL, smoking status, diabetes status, and taking high-blood pressure medication status), JMBayes converged, although its predictive performance was still not comparable to the other tested techniques as shown in Fig. S5.

### Insights from models

**Fig. 3:**
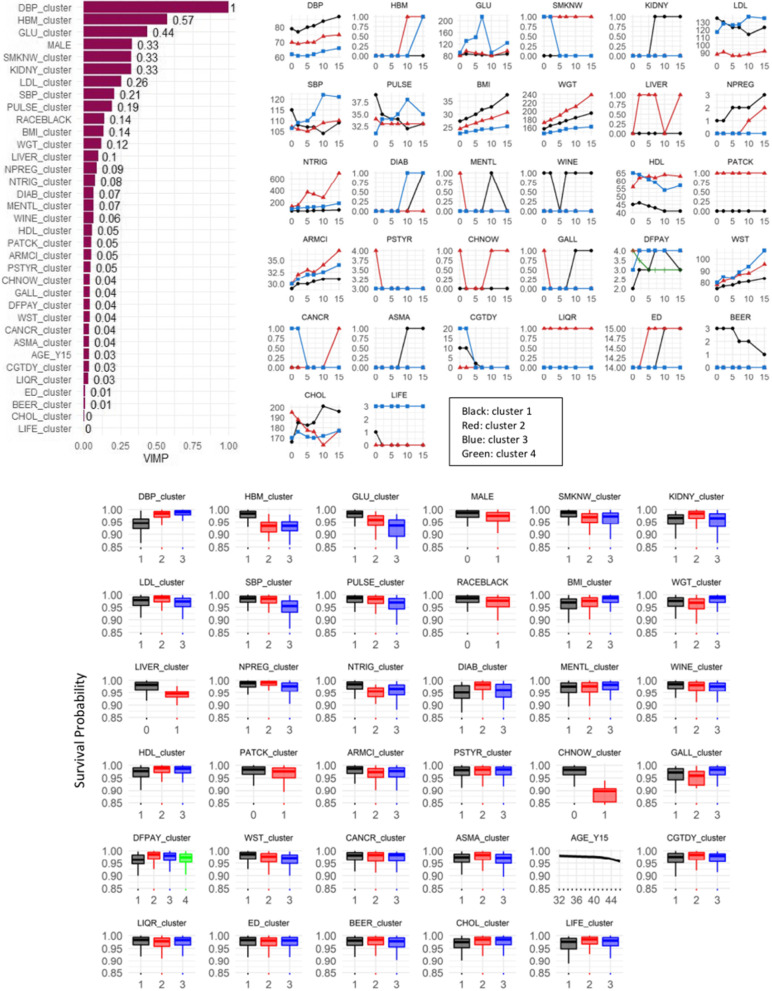
Explanation for the RSF model trained on trajectory clustering data. Top-left panel: normalized median variable importance (VIMP) over 10 folds from permutation for the input variables (trajectory membership and demographic variables) of RSF on trajectory clustering data. Top-right panel: cluster profiles for each longitudinal variable, showing the representative (median) trajectory per cluster. The clustered plots are ordered by the ranking on the left. Bottom panel: partial dependence plots showing the effects of cluster membership (x-axis) on survival probability on the y-axis (1-CVD risk). The colors correspond to the cluster median trajectories in the top panel. DBP: diastolic blood pressure, HBM: taking hypertensive medication (yes/no), GLU: glucose, SMKNW: smoking regularly now (yes/no), KIDNY: kidney problem (yes/no), LDL: low-density lipoprotein, SBP: systolic blood pressure, WGT: weight. The full variable names are explained in the abbreviation section

**Fig. 4:**
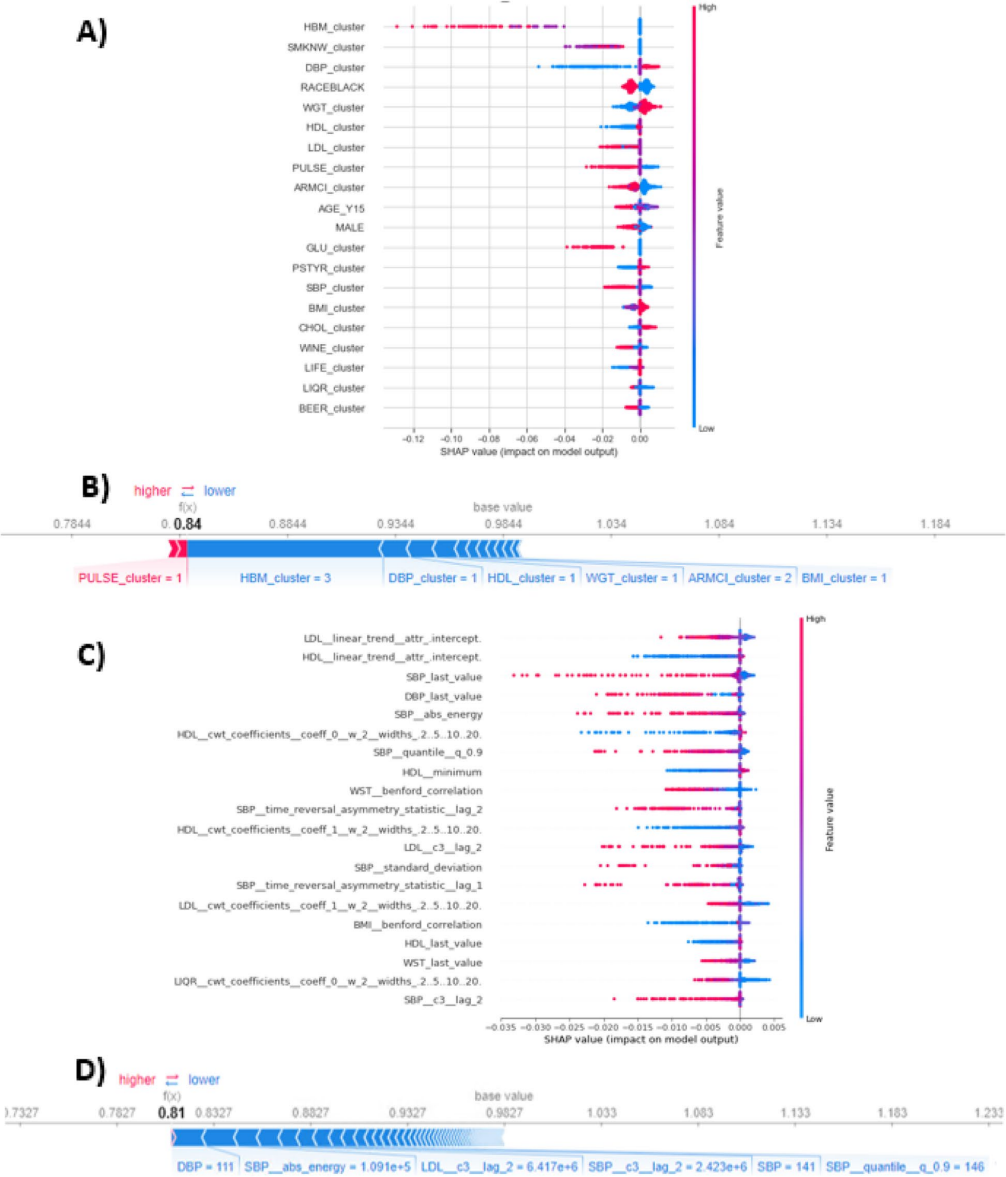
SHAP explanation. Panel A and B: SHAP summary plot for the top-20 predictors of all subjects (**A**) and individual force plot of a single subject (**B**) for RSF model trained on trajectory clustering memberships. High feature value = cluster group 3, low feature value = cluster 1. Panel **C** and **D**: summary plot of the top-20 predictors (**C**) and individual force plot of the same subject (**D**) for RSF trained on 250 time-series extracted summary statistics. In the summary plots, each dot represents a subject. A dot’s position along the x axis (i.e., the actual SHAP value) represents the impact that feature had on the model’s output for that subject, and in this case corresponds to the survival probability (i.e., a lower SHAP value indicates a lower survival probability, or a higher CVD risk). Features are ordered along the y axis based on the mean of their absolute Shapley values. The dot’s color represents high (pink) or low (blue) value of the feature, and dots “pile up” along each feature row to show density. In the individual force plots, a longer arrow indicates a greater impact on pushing the predicted survival probability of the subject towards (pink) or away from (blue) the population average

**Fig. 5:**
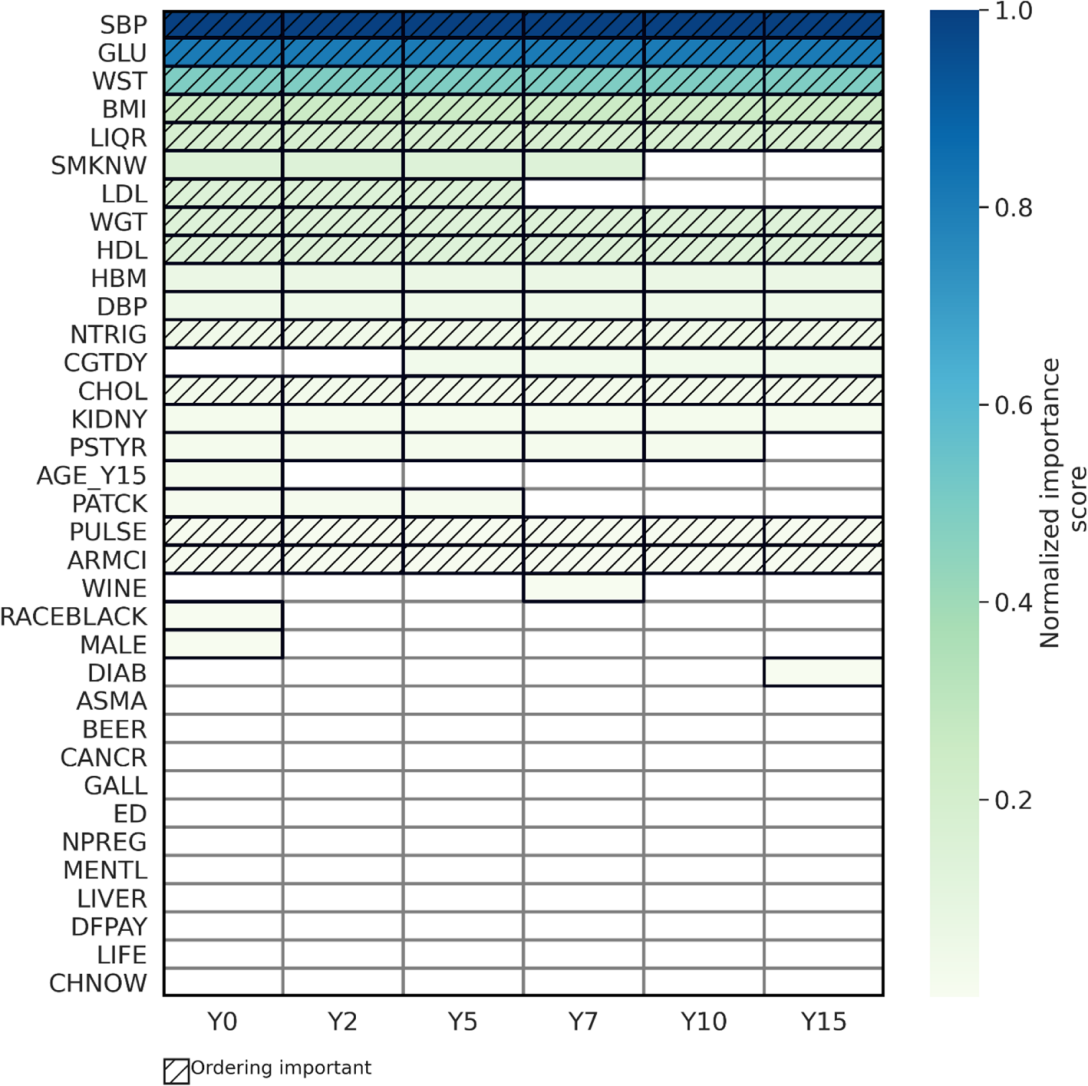
Model explanation of the best performing model, RSF trained on time series extracted features, using TIME. TIME (Temporal Importance Model Explanation) is a model-agnostic longitudinal explanation method. A cell (box) is colored if it’s important, is white if not deemed important by the model. Each row is a variable and shows the most important windows to the model (groups of cells in the same shade of color). The variables are ordered along the y-axis based on the overall importance (darker color = more important). Hatched texture implies the ordering within the window is important to model prediction (i.e., shuffling the variable values at different times within the window affects the model prediction)

We first present the results of the most well-known explanation method, RSF-VIMP, on the most interpretable model, RSF trained on trajectory clustering data (Fig. 3), followed by SHAP results on RSF trained on trajectory clustering data and the best performing model (RSF-TS) (Fig. 4), and finally the results of using TIME to explain the best performing models (Fig. 5). We also attempted to explain the RSF model trained on concatenated data (Fig. S6). Additionally, results for race-specific models are shown in supplemental Table S3 and Fig. S7.

The RSF trained on trajectory clustering data (referred to as RSF-Traj from now on) was an attractive model to explain since it consisted of only the assigned clustering memberships as input and had relatively high AUCs in the early years of the prediction window. Figure 3 shows the explanation for this model, including the variable importance ranking of the input variables, the cluster profiles for each variable, and the partial dependence plots showing the effects of cluster memberships on survival probability. The variable importance ranking was obtained from RSF-VIMP as the average over 10 rankings corresponding to 10-fold cross-validation. The top predictors included trajectories of blood pressure (DBP, SBP), taking anti-hypertensive medication, measures of glucose, smoking status, kidney problem, low-density lipoprotein cholesterol (LDL), as well as gender. Trajectories of BMI and weight also contributed to a degree in the prediction. Regarding the cluster profiles, for continuous variables, the clusters seemed to be separated based on the magnitude, trend, or a combination of both. For example, LDL clusters consisted of low LDL, high-decreasing LDL, and high-increasing LDL. For categorical variables, clusters were separated based on switching state status (e.g., for smoking: always non-smoker, always smoker, and changing from smoker to non-smoker) and time of switching state for categorical variables (e.g., for taking anti-hypertensive medication: always not having to take, start taking at Y5, and start taking at Y10). Regarding relationships with the outcome, high DBP and use of hypertension medications from a young age, high and fluctuating glucose over 15 years, being male, a long history of smoking, high and increasing LDL, steeply increasing SBP and heart rate, high and increasing BMI and other anthropometric measurements (weight, waist girth, arm circumference), having a liver problem, steep increases in triglyceride levels, constantly low HDL, having a parent history of heart attack, taking cholesterol-lowering medication, and economic status (inability to provide the basics for self or family), were all associated with lower survival probability (higher CVD risk). The results of the demographic variables were also consistent with prior knowledge: male, Black, and older age were associated with a higher CVD risk.

Figure 4 shows the SHAP explanation for the same RSF-Traj model and the best-performing model, the RSF-TS model. The SHAP summary plot for RSF-Traj shows how different values of clustering membership in each predictor impacted the SHAP value and the survival probability. A further deviation from the right side indicates a stronger association with a lower survival probability. The predictors with the strongest association with lowered survival probability included HBM, smoking status, DBP, glucose, LDL, HDL, SBP, and pulse beats. In terms of predictor values, being in clusters 2 and 3 for taking anti-hypertensive medication (been taking medication since Y10 or Y5 Exam), cluster 2 for smoking (regular smoker), cluster 1 for DBP (high-increasing), cluster 3 for glucose (high), cluster 3 for LDL (high-increasing), cluster 1 for HDL (low-stable), cluster 3 for SBP (high-rapidly increasing), and cluster 3 for pulse beat (increasing) were all associated with lower SHAP value and lower survival probability.

The force plot for RSF-Traj in Fig. 4 explains why a particular participant of interest has a lower predicted survival probability than the population. Despite their pulse trajectory in the low-stable group, the contributing factors that push the prediction towards higher risk included the fact that they had been taking BP medication for at least 5 years before the last time point, their DBP trajectory was in the high-increasing group, their HDL trajectory was low and slightly decreasing, and the trajectories of their obesity indicators (weight, arm circumference, and BMI) were in high and increasing groups.

The SHAP summary and individual force plots for the RSF-TS model could also be interpreted similarly. The top predictors in RSF-TS included the last observed value of SBP and DBP, SBP absolute energy (sum of all SBP measures from the time series), and intercepts of the linear trend in LDL, HDL. Higher values of linear trend intercept in LDL, last value of SBP and DBP, absolute energy of SBP, and lower values of linear trend intercept in HDL were associated with lower survival probability. Regarding the force plot for RSF-TS, the most impactful contributors to their predicted survival probability being lower than the population average were their last observed DBP value being 111 mmHg, followed by their high sum of all SBP measures, their high c3 statistic value in LDL and SBP (meaning their LDL and SBP trajectories being non-linear, asymmetrical, and increasing), their last SBP value of 141, and followed by other features with decreasing impacts.

Since the inputs of RSF-TS were 250 mathematical time series statistics derived from longitudinal data even after pruning, interpreting a ranking of input variables from SHAP or RSF-VIMP would require extensive knowledge of the mathematical theory behind each statistic, which would not be efficient nor helpful to clinicians. Therefore, here we proposed a more intuitive explanation using the TIME method (Fig. 5). According to Fig. 5, the most important variables were SBP, Glucose, and Waist Girth (> 0.5 normalized importance score); followed by BMI, Amount of Hard Liquor Drink Per Week, Weight, HDL, Taking Anti-hypertensive Medication, DBP, and Triglycerides, with the important windows covering the entire length, indicating all time steps were important. Regarding demographic variables, only the first value of age, race, and gender was deemed important. The order within the important windows was important to model prediction in most variables, except for taking BP medication, DBP, physical activity, and kidney problems. TIME also showed that early measurements in some variables were more important for lifetime CVD prediction than the more recent measurements, such as smoking, LDL, and parent history of heart problems.

## Discussion

There is limited work in the literature that incorporates longitudinal data for risk prediction using survival analysis and little comparison of recently introduced methods and statistical methods in terms of their predictive accuracy and utility in cardiovascular medicine. Our study aims to fill these gaps by investigating six strategies spanning a wide range of different methodologies for incorporating multivariate longitudinal data into long-term CVD prediction in young adults. Our study presents a comparative, interpretable, and scalable ML framework for survival analysis using multivariate longitudinal data. We provide a thorough discussion on spatial (variable) and temporal interpretability, as well as epidemiological utility, to fill the gap left by the majority of ML works for longitudinal data, which only focus on predictive accuracy. We also emphasize that the decision to pick the best methodology should be guided by both interpretability and predictive performance. Our study demonstrates the application of the proposed framework for predicting CVD in young adults using longitudinal data. Young adults are often underrepresented in research, but understanding their cardiovascular risks at a young age could have a big impact on their life course. Our interpretability analysis of risk factors’ trajectory history provides valuable epidemiological insights for young adults, including the cumulative and irreversible effects of early exposure to risk factors on lifetime cardiovascular development.

### Added predictive value of longitudinal data

One of the goals of our study was to answer the question of what is the added value of longitudinal data. In terms of predictive performance, we show that longitudinal data improved up to 8% in AUC and C-index, compared to using baseline values alone, and up to 4% compared to using the last observed data. The integrated AUCs over 17 years show that there is a small difference between the models using longitudinal history versus the best model using the last observed data (+ 0.016), but the integrated AUCs for post-10-year CVD risks show a clearer difference (+ 0.028). These results suggest that most of the predictive value of using longitudinal data lies in the long-term, whereas short-term prediction could use either data from multiple time points or just the last time point. In the literature, the number of papers reporting the difference between with and without the incorporation of repeated measurements is limited. Among those that report, the added improvement in C-index is 0.072 (confidence interval 0.002–0.139) on average, as shown in a structural review in the critical care setting [25]. Another report notes that JM for SBP and DBP leads to a gain of 0.03–0.04 in AUC [2]. The added improvement observed in our study is within these ranges.

Regarding the comparison of modeling strategies, in terms of predictive performance, the results suggest that the time series massive summary statistics extraction performs the best in both discrimination and calibration. The DL-based survival method Dynamic-DeepHit and the data concatenation method had a slightly lower performance. This finding agrees with previous reports showing trend analysis from the time series is more important in discriminating cases from controls than just using the raw time series as inputs in predicting cardiac arrest [55, 56]. However, these studies are classification problems, our study extends the application of time series massive feature extraction to survival analysis. We believe that the superior performance of this strategy comes from the extracted time series features that cover many characteristics from the time series such as trend, symmetry, abrupt transitions, number of peaks, etc., and thus help preserve longitudinal information at different levels of granularity. These features, coupled with a method robust for high-dimensional data like RSF, lead to the best predictive performance.

The trajectory clustering strategy has the highest median AUC compared to the other algorithms up to 10 years of follow-up, however, the performance drops further into the prediction window (Fig. 2). Few papers have used trajectory clustering as input to survival analysis before, but they are limited to clustering one or two covariates [32, 57]. We extend this strategy to a higher-dimensional setting. This approach could be thought of as a dimensionality reduction alternative to the time series feature extraction approach. Trajectory clustering does not increase the dimension of the data at all since the only input to the survival model is the cluster membership. Of course, because of this, this approach is unable to retain fine-grained information but still maintains a respectable predictive performance, and it might be easier to interpret for clinicians and explainable to patients.

Interestingly, the data concatenation strategy, which includes all past measurements and treats repeated measures as independent input variables, performs relatively well. Its discriminative metrics are worse than the time series extraction approach but better than just using the last observed values. This approach has been employed in several works before and shown to perform just as well as, if not better than, using one time-point [13, 39, 40]. If the end goal is to look only at predictive capability, this approach’s simple underlying idea, straightforward implementation, and ability to include all past measurements could make it an attractive approach.

It is also interesting to note that JM is among the worst-performing strategies. It did not converge when the number of covariates with repeated measures was high (32), and when the number was low (9), its predictive performance was the lowest among all strategies. In the literature, despite the growing number of studies adopting JM for a dataset, few works compare JM with a variety of other longitudinal methods. The observations from our study agree with previous works showing JM is no better than using just the last observed values [5, 39], and not as good as the dynamic DL survival method Dynamic-DeepHit [22]. It might be worth noting that in our experiments with JM, we did not exhaustively try all tuning options, for example, we did not experiment with different specifications of the functional form linking the longitudinal and survival processes and instead used the default form, which adds the participant-specific linear predictors of the mixed-effect models as time-varying covariates in the survival model. However, changing the functional form has been shown to only offer a modest increase, if any, or even lowers the AUC [58]. In addition, since the survival model of JM is based on Cox and the longitudinal sub-models are linear mixed-effect models, they share the same limitations as Cox and mixed models, such as the inability to handle many covariates, non-linearity, and proportional-hazard assumption. Because of these reasons and findings, further comparative studies are needed to advocate the value of JM in higher-dimensional settings.

Another interesting observation is when the number of covariates is small, complex methods like time series massive feature extraction or Dynamic-DeepHit perform no better than Cox trained on the last observed values, as shown in our experiment using only 9 ASCVD risk factors, of which 6 are longitudinal covariates (Fig. S5). This finding agrees with some previous reports using a small number of longitudinal covariates for CVD prediction, observing that longitudinal data only offers modest improvement or no improvement at all compared to just using data from one time-point [5]. This result suggests that the predictive value of longitudinal data shines in a higher-dimensional setting, although further research with varying data dimensionalities is needed to confirm this observation.

Overall, all strategies using longitudinal data perform better than using cross-sectional data at baseline. Among the strategies, the last observed value strategy has the worst discriminative performance in the higher-dimensional setting, while the time series massive feature extraction is the best, followed by dynamic DL survival, trajectory clustering, and concatenation. In terms of calibration, most strategies have similar Brier Scores, with the time series feature extraction strategy having the lowest one.

### Temporal model interpretability

While the improvement in predictive capabilities is one reason to use longitudinal data, the other is to identify disease processes both at the population and the individual level using trends and outliers in data, that cross-sectional data often misses. Oftentimes, the quantification of predictive improvement is performed using just the differences in C-index which offers limited clinical interpretability, as noted by [24]. Decreased interpretability and limited clinical value are two main reasons that limit the adoption of longitudinal data in prediction modeling [25]. Interpreting models using longitudinal data is even more challenging than those using tabular data (where each variable takes a single value instead of a sequence of repeated measures) since the temporal nature of longitudinal data renders methods that work with tabular data unable to work with raw time series. For example, permutation-based explainability methods for tabular data would carry permutations of individual timesteps in the temporal setting, which ignores the temporal structure of the data and correlations within the time series [54]. In this work, we proposed and examined two solutions to this problem: (1) summarizing the information from longitudinal data so that the input for the survival model is in tabular format, then applying explainability methods for tabular data, and (2) directly using explainability methods specifically designed for temporal data. The first solution worked conveniently with the time series massive feature extraction strategy and the trajectory clustering strategy, since they essentially represent the time series in summary statistics, and thus we explained models adopting those two strategies with a model-specific explainability method, RSF-VIMP, and a model-agnostic one, SHAP. For models adopting strategies that take in raw time series as input, only the second solution would work, and we used the model-agnostic TIME method to explain those models.

The following structure of this sub-section is as follows: we first discuss the results and implication of the most well-known method, RSF-VIMP, on the most interpretable model, RSF-Traj, followed by SHAP on RSF-Traj and RSF-TS, then followed by TIME.

#### RSF-VIMP

As shown in the Results section, RSF-VIMP on RSF-Traj is perhaps the most interpretable and simple of all. RSF-VIMP is one of the most well-known methods. The information from trajectory clustering is simple as it only includes clustering memberships. Coupling the global importance ranking from VIMP with a visualization of the cluster profiles and the partial dependence plots gives the clinician a comprehensive picture of which subgroups are high- or low-risk for CVD. The model explanation identifies and confirms the association of trajectory groups with CVD risks observed in some previous trajectory modeling works that focused on a specific risk factor, such as high BP trajectories throughout young adulthood, associated with increased CVD risk in middle age [4, 59]. The rapid increase BMI trajectory group is associated with a higher risk of CVD compared to the other BMI groups, which aligns with previously reported observations in BMI trajectories [60]. The trajectories for indicators of metabolism, including triglyceride, LDL, HDL, total cholesterol, and glucose, also show that high or increasing groups are associated with higher CVD risks, which agrees with the result from a previous CARDIA study showing high association of initially high or worsening metabolic trajectories with greater prevalence and extent of coronary artery calcification and myocardial dysfunction [33]. In the epidemiological setting, we envision that the trajectory clustering model along with its explanation and visualization could help clinicians and epidemiologists identify subsets of participants with distinct trajectory profiles, select high-risk groups, and better understand the impact of temporal evolution of risk factors to heart disease, which could be helpful in planning preventive strategies.

#### SHAP

Unlike RSF-VIMP which is limited to RSF, SHAP works with any algorithm that can provide a mapping function from the input matrix to the predicted outcome. There have been discussions that investigate and compare how different ML algorithms treat tabular data differently using SHAP [61], but our study, to our knowledge, is among the first to apply SHAP to explain information extracted from longitudinal data for a time-to-event outcome. The added value of the SHAP summary plot compared to VIMP’s variable ranking is that SHAP displays how different feature values impact the model’s predicted survival probability and quantifies the degree of impact on the x-axis. Furthermore, the SHAP individual force plots offer a unique personalized explanation to a participant of interest, explaining how the participant’s feature values contribute to the model’s predicted survival probability, as demonstrated in the Results.

Regarding the individual explanation between the trajectory clustering strategy and the massive feature extraction strategy, the former appears to be more intuitive than the latter. On the same participant of interest in Fig. 4, both trajectory clustering and time series feature extraction assign a lower and similar survival probability than the population means in both strategies. However, the trajectory clustering points out that, the reasons for the participant’s lowered predicted survival probability include their history of taking BP medication, their high-increasing DBP trajectory, low-decreasing HDL trajectory, and high-increasing trajectories of obesity indicators (weight, arm circumference, and BMI). The time series feature extraction, on the other hand, relies on many summary statistics to predict. The number of the contributing factors is high with the most impactful predictors being summary statistics of SBP and LDL (last observed DBP and SBP, SBP absolute energy (sum of all values), statistics indicating non-linearity and increasing trend in LDL and SBP, and many more factors). This analysis of individual explainability suggests that SHAP works better with few and interpretable inputs like those provided from trajectory clustering. Another way of interpretation is needed to better explain methods with high-dimensional, mathematical-complex input like RSF-TS. That led us to use TIME.

#### TIME

TIME is one explainability technique that explains RSF-TS better, along with the capability of explaining all temporal models using raw time series as input. TIME is one of the only truly model-agnostic interpretability methods introduced to date, with very limited alternatives. Most of the limited number of proposed methods for temporal model explanation are model-specific methods, like saliency maps and class activation maps, that only work with certain neural network architectures [62–65]. Some recently prototyped methods that appear to be model-agnostic, such as FIT and Win-IT [26, 66], work with neural networks only. In this paper, we show TIME provides a simplified and intuitive explanation for the best-performing model, RSF-TS, more than SHAP does. The output for TIME is a heatmap with the rows being the unique variables and the columns being time steps, regardless of the transformation done on the trajectories. Besides providing a global importance variable ranking, TIME shows the window of importance for each variable and whether the ordering within the window is important.

One interesting finding is that in some time-varying variables, the important window was in earlier time steps than in later time steps. To be specific, TIME identified three variables of which measurements in early adulthood were more important for lifetime CVD prediction than those in the middle-age stage in this cohort: smoking status, LDL, and parent history of heart problems. These findings on smoking and LDL suggest that the presence of cumulative effects from a young age may cause irreversible organ damage and permanently elevates long-term CVD risk, regardless of the change in those risk factors at an older age. Several reports support these findings, such as long-term smoking may cause irreversible arterial stiffness, and passive exposure to cigarette smoke since childhood might cause irreversible damage in endothelium-dependent vasodilation [67]. For LDL, the importance of LDL in early adulthood for long-term CVD risk is plausible since it is a major pathogenic contributor to atherogenesis and a marker of endothelial dysfunction [68]. The effect of LDL cholesterol on the risk of CVD has been stated to be both causal and cumulative over time [69]. Parent’s heart problem at the participant’s young age is plausible as genetics could be an attribution to the participant’s CVD risk, while CVD problems at the old age could be due to many environmental and lifestyle factors. Regarding other important variables, TIME noted the information across all time points from young adulthood to the last observed values in DBP, SBP, Glucose, BMI, waist girth, and lipids (cholesterol, triglycerides, and HDL), highlighting the importance of cumulative effects of these risk factors in long-term CVD prediction for young adults. TIME is particularly suited in studying trends at the level of the population.

Overall, we demonstrate how different model explainability methods work to explain ML survival models incorporating longitudinal data. Trajectory clustering coupled with RSF-VIMP and SHAP provides the most intuitive explanations, while TIME is suitable for models using raw time series as input or using complex feature engineering as input.

### Limitations and future directions

A limitation of our study is not being able to cover many methods and different variations within each modeling strategy. For example, there are several methods in JM, such as the joint latent class model and joineRML [70, 71], and DL longitudinal survival methods such as MATCH-Net [23]. With the growing new methods introduced, it can become intractable to include all methods. However, for this comparative study, we tried to pick one representative method from each modeling strategy, usually from the most cited papers. Another limitation is we do not have an external validation as CARDIA is a unique cohort with 30 years of follow-up, and our study would benefit from validation in other large cohorts of younger-aged individuals. The number of CVD events by the end of follow-up was relatively small. In addition, the CARDIA study consists of Black and White participants in the US with baseline data collected in 1985, and thus the results from this work may not be transferable to other populations of different demographic characteristics. Future direction of this work includes investigating dynamic prediction, which updates the participant’s CVD risk with new data information. JM and Dynamic-DeepHit are most suitable for dynamic prediction, and we did carry dynamic prediction using Dynamic-DeepHit in the Supplement, but the other modeling strategies would require training new models at every landmark time and thus would complicate the analysis. Competing risks or outcomes with recurrent events are also outside of the scope of this study. Lastly, it is important to note that explainability methods like RSF-VIMP, SHAP, and TIME do not imply causality. Another future direction of this work is to explore and validate the usability of this work in clinical settings.

## Conclusion

In conclusion, we demonstrate the added value of multivariate longitudinal data in predictive accuracy and epidemiological utility in CVD prediction in young adults both at the population and individual levels. Using a unified framework that evaluates and compares model performance and explainability in six different strategies of analysing longitudinal data, we argue that spatial and temporal interpretability should be emphasized when opting for a method. The trajectory clustering approach coupled with RSF provides the most intuitive explanations while still maintain comparatively good predictive performance. The comparative interpretability analysis reveals insights about the effects of the risk factors’ trajectory history on cardiovascular risk development in young adults. Our framework could be extended to a higher number of variables and other methods dealing with repeated measures to better utilize longitudinal data.

## Supplementary Information

**Additional file 1.**

## Data Availability

The CARDIA dataset can be requested via the study website https://www.cardia.dopm.uab.edu/. CARDIA study data are available to affiliated and non-affiliated investigators. See the study website for further details: http://www.cardia.dopm.uab.edu/invitation-to-new-investigators. R packages used in this study (*traj, NbClust, JMBayes, rsfsrc*) are publicly available on CRAN (https://CRAN.R-project.org). Python packages are accessible on Github (*TIME*: https://github.com/cloudbopper/anamod; *SHAP*: https://github.com/slundberg/shap; *Dynamic-DeepHit*: https://github.com/chl8856/Dynamic-DeepHit; *tsfresh*: https://github.com/blue-yonder/tsfresh).

## Abbreviations

CVD: cardiovascular disease
DL: Deep Learning
iAUC: integrated AUC
JM: joint modeling
JMBayes: Joint modeling with Bayesian approach
LASSO-Cox: Cox Proportional Hazards penalized by Least Shrinkage and Selection Operator
ML: machine learning
RSF: Random Survival Forest
RSF-Traj: Random Survival Forest trained on trajectory clustering data
RSF-TS: Random Survival Forest trained on time series massive extraction features data
SHAP: Shapley Additive explanation
TIME: Temporal Importance Model Explanation (TIME)
VIMP: Variable Importance

## Variable encoding

ASMA: status of having asthma
ARMCI: arm circumference
BEER: Number of drinks of beer per week
BMI: Body Mass Index
CANCR: status of having cancer
CGTDY: number of cigarettes/day
CHNOW: taking cholesterol-lowering medication
CHOL: Cholesterol
DBP: diastolic blood pressure
DIAB: status of having diabetes
DFPAY: ability to pay for the basics
ED: education
GALL: gallbladder problem
GLU: glucose
HBM: taking anti-hypertensive medication
HDL: high-density cholesterol
KIDNY: kidney problem
LDL: low-density cholesterol
LIFE: Number of times taking marijuana in life
LIQR: Number of hard liquor shots per week
LIVER: liver problem
MENTL: mental disorder
NPREG: times being pregnant
NTRIG: triglycerides
PATCK: Parent history of heart attack
PSTYR: Self-assessed physical activity level
PULSE: pulse beats
SBP: systolic blood pressure
SMKNW: Still smoking regularly (>=5 times/week)
WGT: weight
WINE: Number of drinks of wine per week
WST: waist girth
