# Supplementary material for: Multivariate longitudinal data for survival analysis of cardiovascular event prediction in young adults: insights from a comparative explainable study
Source: BMC Med Res Methodol. 2023 Jan 25;23:23. doi: 10.1186/s12874-023-01845-4 (PMC9878947; doi:10.1186/s12874-023-01845-4)
Supplement: Supplementary file 1 — Additional file 1. [file 12874_2023_1845_MOESM1_ESM.pdf]

SUPPLEMENTARY MATERIALS

Multivariate longitudinal data for survival analysis of cardiovascular event prediction in young adults: insights from a comparative explainable study

List of supplementary figures and tables

Table S1. A list of the variables that were used for prediction in this study

Fig. S1: cumulative incidence of CVD after Y15 and Y5

Fig. S2: cohort selection flowchart

Fig. S3: performance among 23 clustering criteria to select the optimal cluster assignment for the trajectory clustering strategy

Fig. S4: Time-varying AUC on the test set using Dynamic-DeepHit for dynamic prediction on all participants in CARDIA.

Table S2: Model performance in additional metrics at the last evaluation time point

Fig. S5: Model performance over time when limiting the input variables to 9 traditional ASCVD risk factors

Fig S6: heatmap showing variable importance for the RSF model trained on concatenated data

Table S3: Race-specific model predictive performance for the top longitudinal modeling strategies

Fig. S7: Explanation for race-specific models of RSF trained on trajectory clustering data.

Outcome ascertainment

The CARDIA study outcomes ascertainment protocols have been described in detail elsewhere [1]. For this study, the first CVD event was used as the endpoint [2, 3]. We recorded new cardiovascular and cerebrovascular events from the baseline examination through August 2018. During their scheduled study examinations and yearly telephone interviews, each participant or designated proxy was asked about interim hospital admissions, outpatient procedures, and deaths. Designated proxies do not participate in the examination. Medical records were requested for participants who had been hospitalized or received an outpatient revascularization procedure. Vital status was assessed every 6 months; medical and other death records were requested after consent had been obtained from the next of kin. Two physician members of the Committee independently reviewed medical records and recorded information to adjudicate each possible cardiovascular or cerebrovascular event or underlying cause of death using specific definitions and a detailed manual of operations (available online: http://www.cardia.dopm.uab.edu). If disagreement occurred between the primary reviewers, the case was reviewed by the full committee. The primary composite outcome was incident CVD, which included coronary heart disease (CHD – myocardial infarction, acute coronary syndrome, or CHD death, including fatal myocardial infarction), stroke, transient ischemic attack (TIA), hospitalization for heart failure, intervention for peripheral arterial disease, or death from cardiovascular causes. Secondary cause-specific outcomes included stroke/TIA, CHD, and CVD mortality. Participants who died from a non-CVD cause were censored in the survival models at time of death.

Temporal Importance Model Explanation (TIME)

Here, we briefly summarize the algorithm of TIME in layman's terms, for a more detailed technical version please refer to [4]. The underlying working of TIME is its permutation approach.

A typical way of permutation in tabular format is to replace the value of feature j in participant i with another value of j in another participant, then compute the difference between the permuted and baseline losses. The baseline loss is the different between the model output and the target outcome y_i, and the permuted loss is the difference between the model output using the permuted input and the target outcome y_j. If the permuted loss is significantly greater than the baseline loss

on average over many permutations, the feature is deemed important. For the case of longitudinal data however, the typical permutation would be simply replacing the value of feature *j* at time *t* in participant *i* with another value of *j* at time *t* in another participant. Doing this would break the temporal dependencies and correlations within the trajectory as noted above. To alleviate this problem, TIME performs joint permutation, which means (1) replacing values of feature j from a time window in participant i with the values in another participant of the same time window, instead of individual time points, and (2) replacing the value of feature j from time k1 with that of feature j from time k2, from the same participant, which enables ordering importance.

As for the time window, TIME searches for the most important time windows W* = [k1, k2] (1<=k1<k2<=L) that most of the effect of permuting lies in W* (L is the length of time series, in this work is 6). TIME does this by searching for the largest possible prior window W_P = [1, k1] and subsequent window W_S = [k2, L]. TIME initializes W_p to be the first half and W_S be the latter half of the series, then perturb W_P and W_S and observe their importance scores. If the importance score for W_P is high, it likely that W_P contains important time steps, the search algorithm will shorten the W_p to exclude the important time steps, and if the importance score for W_P is low, W_P will then be expanded until its importance score is greater than a threshold. This threshold of importance is determined from a user-input localization parameter that specifies the level of importance that the importance window should hold (for example, 90% of the total importance of the whole series). Similar logic is applied to find the subsequent window W_S, and the important window W* is what in between W_P and W_S.

Another attractive feature of TIME is using hypothesis testing with correction for multiple comparisons, using the permutation test [5] to ascertain importance at three levels: overall (global), window, and ordering within the window, for each longitudinal variable. TIME uses a hierarchical false discovery rate control method [6] to address the issue of multiple comparisons in hypothesis testing.

References:

1. Friedman GD, Cutter GR, Donahue RP, Hughes GH, Hulley SB, Jacobs DR, Liu K, Savage PJ. Cardia: study design, recruitment, and some characteristics of the examined participants. *J Clin Epidemiol*. 1988;41:1105–1116.
2. Loria CM, Liu K, Lewis CE, Hulley SB, Sidney S, Schreiner PJ, Williams OD, Bild DE, Detrano R. Early Adult Risk Factor Levels and Subsequent Coronary Artery Calcification The CARDIA Study. *J Am Coll Cardiol*. 2007;49:2013–2020.
3. Gardin JM, Wagenknecht LE, Anton-Culver H, Flack J, Gidding S, Kurosaki T, Wong ND, Manolio TA. Relationship of Cardiovascular Risk Factors to Echocardiographic Left Ventricular Mass in Healthy Young Black and White Adult Men and Women: The CARDIA Study. *Circulation*. 1995;92:380–387.
4. Sood, A., and Craven, M. Feature Importance Explanations for Temporal Black-Box Models. *arXiv preprint arXiv:2102.11934*. 2021.
5. Ojala, M. and Garriga, G. C. Permutation Tests for Studying Classifier Performance. *Journal of Machine Learning Research*. 2010:1833–1863.
6. Yekutieli, D. Hierarchical False Discovery Rate–Controlling Methodology. *Journal of the American Statistical Association*. 2008; 103(481):309–316.

Table S1. A list of the variables that were used for prediction in this study. A total of 35 variables/predictors were used, three of which were fixed variables and in *italic*, the rest were longitudinal (repeating) variables that were repeatedly measured in most (if not all) exams in most participants.

**Category**	**Variable name**	**Variable description**
Demographics	*AGE, MALE, RACE*	Age, male or not, race: African-American or White
Socioeconomic	ED, DFPAY	Education, ability to pay for the basics
Body measures	BMI, ARMCI, WGT, WST	BMI, arm circumference, weight, waist girth
Medical history	ASMA, CANCR, DIAB, GALL, KIDNY, NPREG, LIVER, MENTL, PHRTAK	Asthma, cancer, diabetes, gallbladder problem, kidney problem, number of pregnancies, liver problem, mental disorder, parent's history of heart attack
Alcohol use	BEER, LIQR, WINE	Number of drinks of beer/hard liquor/wine per week
Smoking	SMKNW, CGTDY	Still smoking regularly (>=5 times/week), number of cigarettes/day
Heart measures	DBP, SBP, PULSE, HBM	Diastolic blood pressure, systolic blood pressure, pulse, taking anti-hypertensive medication
Lipids	CHOL, CHNOW, HDL, LDL, NTRIG	Total cholesterol, taking cholesterol medication, high-density cholesterol, low-density cholesterol, triglycerides
Glucose	GLU	Fasting glucose
Marijuana use	LIFE	Number of times taking marijuana in life
Physical activity	PSTYR	Reported participation in 13 physical activities

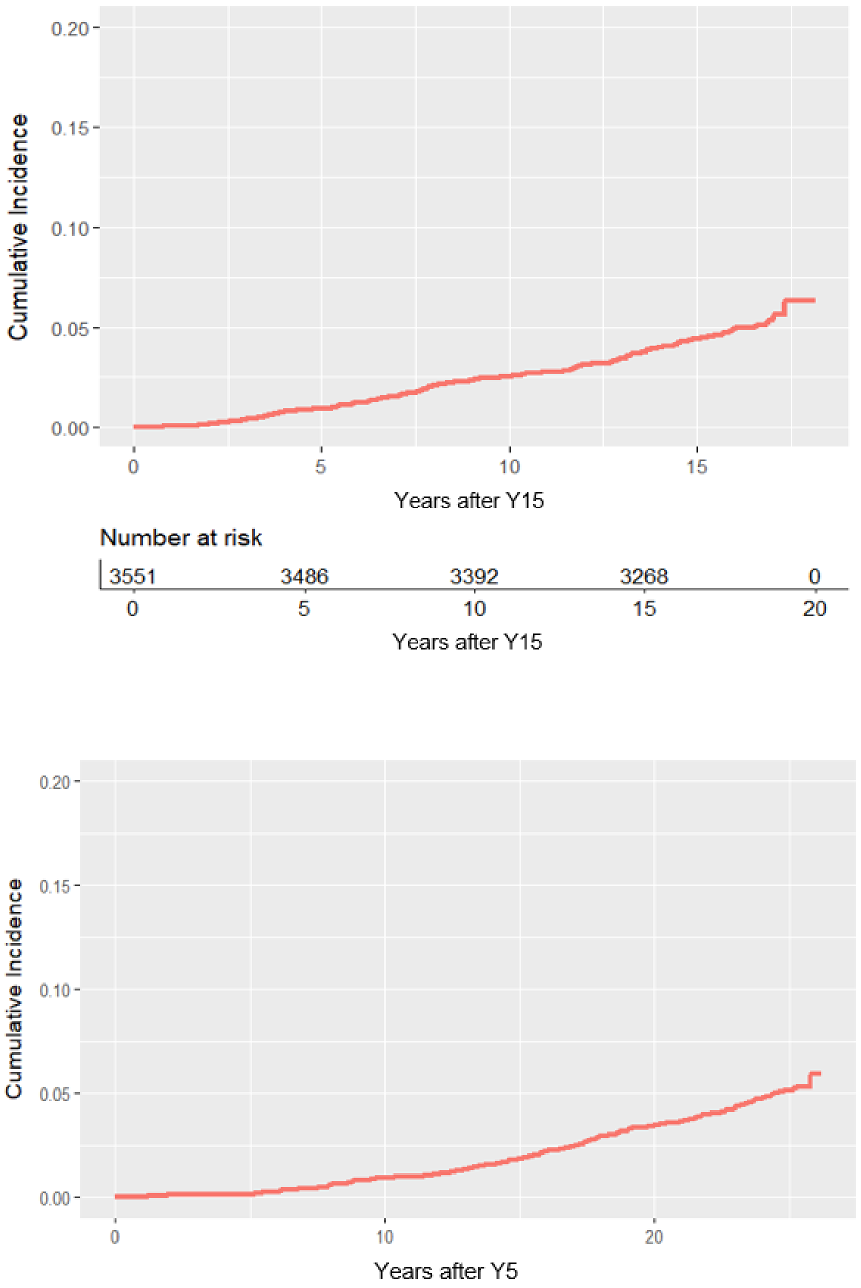

Fig. S1: cumulative incidence of CVD after Y15 (top) and Y5 (bottom). The cumulative incidence could range from 0 to 1 (max). Few incidents happened before Y15, as the curve is relatively flat (very few incidents) before Y15 Exam (10 Years after Y5 Exam). After Y15, the incidence rate is roughly linear.

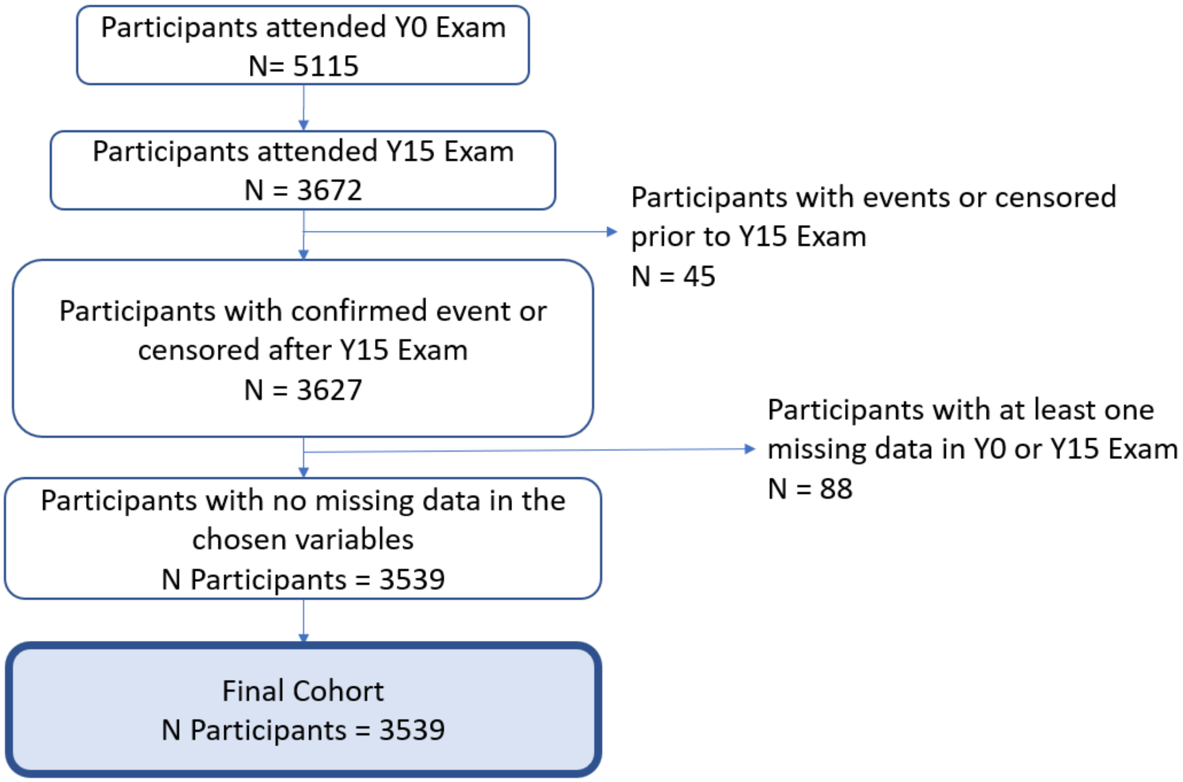

Fig. S2: cohort selection flowchart

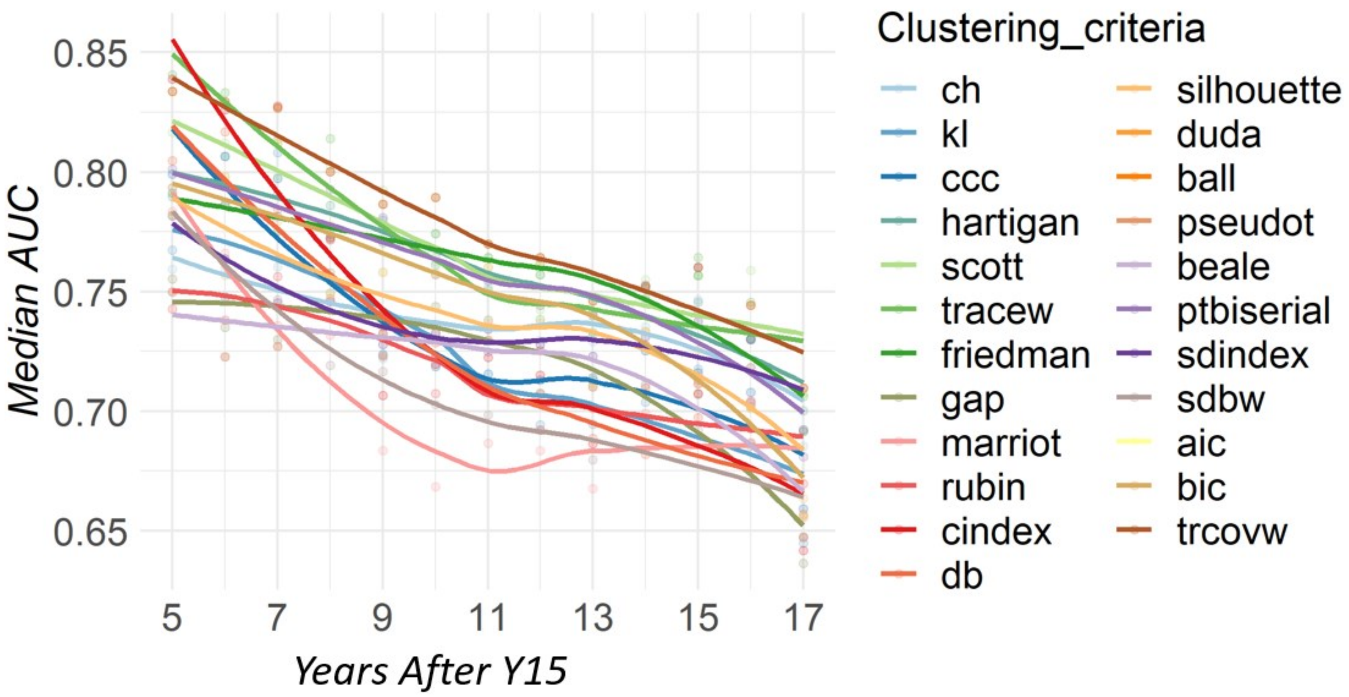

Fig. S3: performance among 23 clustering criteria to select the optimal cluster assignment for the trajectory clustering strategy. The best criterion is 'trcovw' method (in brown), standing for trace (or sum of diagonal elements) of the within-cluster pooled covariance matrix.

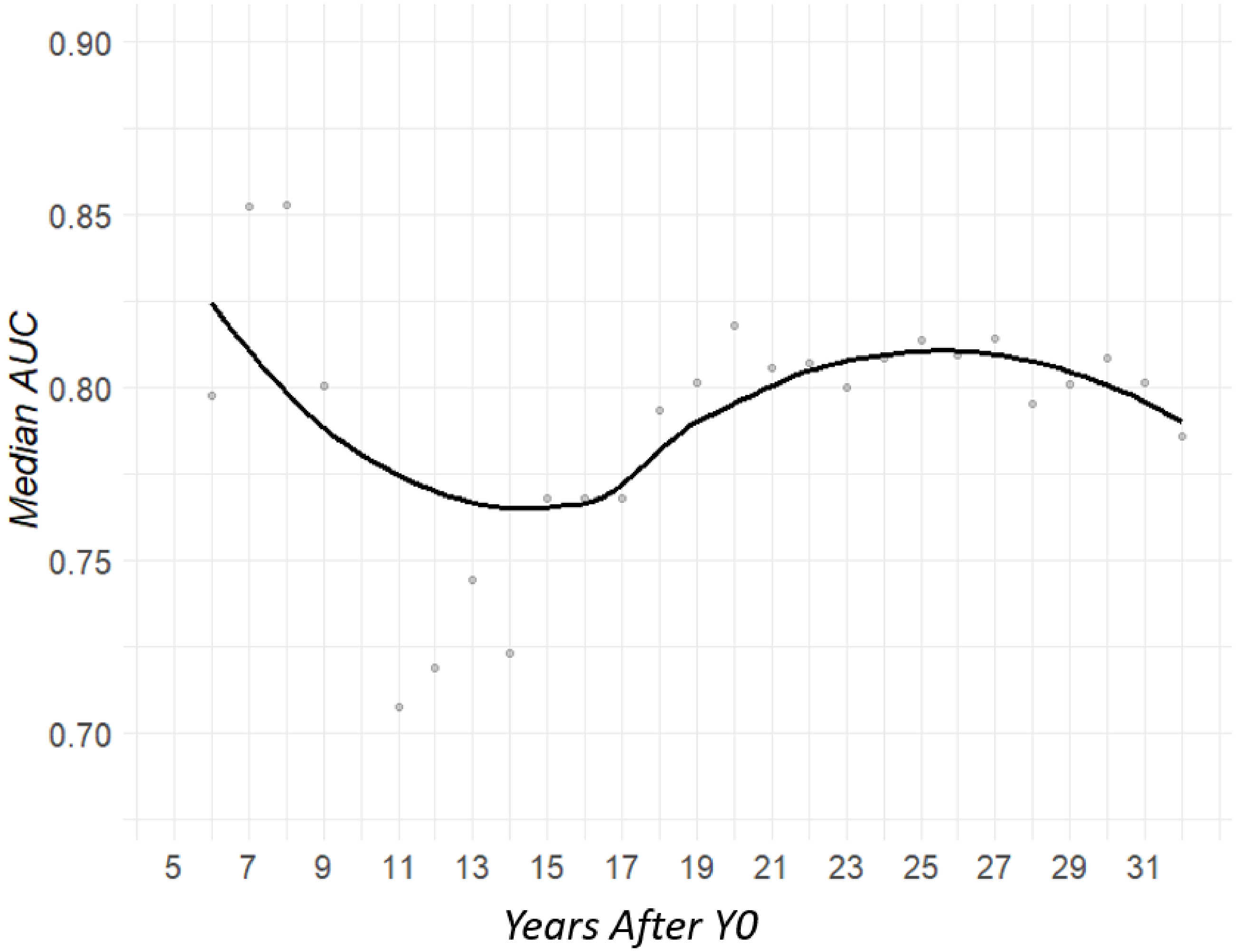

Fig. S4: Time-varying AUC on the test set using Dynamic-DeepHit for dynamic prediction on all participants in CARDIA. The model was trained and validated using 5-fold x 2 times cross-validation. AUC before Y15 is unstable because of the low CVD incidence before Y15.

Table S2: Model performance in additional metrics at the last evaluation time point (17 years after Y15). The binary cutoff threshold is determined by the point on the AUROC curve that maximizes F1 score. Bolded values indicate the highest value in the column.

**Strategy**	**Model**	**Post-10 years iAUC**	**Brier**	**Sensitivity**	**Specificity**	**PPV**	**NPV**	**F1**	**MCC**
Time-series (TS) massive feature extraction	RSF on TS-extracted features	**0.792 (0.773, 0.811)**	**0.042 (0.041, 0.043)**	**0.779 (0.715, 0.842)**	0.653 (0.578, 0.724)	**0.118 (0.093, 0.137)**	**0.982 (0.979, 0.986)**	0.202 (0.169, 0.228)	0.208 (0.175, 0.237)
	LASSO-Cox on TS-extracted features	0.733 (0.7, 0.771)	0.045 (0.044, 0.047)	0.678 (0.616, 0.741)	0.673 (0.612, 0.735)	0.110 (0.094, 0.124)	0.974 (0.971, 0.978)	0.187 (0.166, 0.207)	0.171 (0.145, 0.196)
Recurrent neural network	Dynamic-DeepHit	0.785 (0.756, 0.813)	0.047 (0.046, 0.048)	0.765 (0.698, 0.846)	0.676 (0.603, 0.761)	0.122 (0.107, 0.140)	0.982 (0.978, 0.986)	**0.208 (0.187, 0.234)**	**0.214 (0.186, 0.246)**
Trajectory clustering	RSF on trajectory clustering data	0.778 (0.760, 0.796)	0.043 (0.042, 0.044)	0.758 (0.697, 0.832)	0.611 (0.546, 0.665)	0.101 (0.087, 0.112)	0.977 (0.974, 0.98)	0.176 (0.159, 0.191)	0.979 (0.976, 0.983)
Data concatenation	RSF on concatenated data	0.779 (0.761, 0.798)	0.043 (0.042, 0.044)	0.783 (0.736, 0.824)	0.635 (0.57, 0.697)	0.112 (0.095, 0.127)	0.982 (0.978, 0.985)	0.194 (0.17, 0.216)	0.197 (0.168, 0.227)
Joint modeling	JMBayes			Did not converge					
Last observed values	RSF on Y15 Data	0.765 (0.74, 0.79)	0.043 (0.042, 0.044)	0.684 (0.638, 0.73)	**0.699 (0.651, 0.75)**	0.116 (0.103, 0.129)	0.976 (0.973, 0.978)	0.196 (0.178, 0.215)	0.187 (0.164, 0.209)
	Cox on Y15 Data	0.761 (0.738, 0.788)	0.043 (0.042, 0.045)	0.656 (0.587, 0.732)	0.722 (0.663, 0.777)	0.125 (0.099, 0.144)	0.975 (0.971, 0.979)	0.203 (0.179, 0.225)	0.191 (0.168, 0.219)
	LASSO-Cox on Y15 Data	0.762 (0.752, 0.802)	0.044 (0.043, 0.045)	0.698 (0.65, 0.749)	0.685 (0.642, 0.726)	0.114 (0.102, 0.126)	0.976 (0.972, 0.98)	0.195 (0.178, 0.211)	0.186 (0.164, 0.206)
Reference (Y0 data)	RSF on Y0 Data	0.737 (0.71, 0.762)	0.044 (0.044, 0.045)	0.662 (0.592, 0.729)	0.669 (0.631, 0.713)	0.100 (0.094, 0.106)	0.974 (0.969, 0.978)	0.173 (0.163, 0.183)	0.156 (0.137, 0.176)
	Cox on Y0 Data	0.738 (0.711, 0.764)	0.045 (0.044, 0.046)	0.696 (0.608, 0.784)	0.635 (0.552, 0.724)	0.102 (0.084, 0.115)	0.976 (0.971, 0.98)	0.174 (0.154, 0.19)	0.16 (0.141, 0.177)
	LASSO-Cox on Y0 Data	0.729 (0.704, 0.757)	0.045 (0.044, 0.046)	0.629 (0.569, 0.692)	0.688 (0.632, 0.756)	0.104 (0.095, 0.113)	0.972 (0.97, 0.974)	0.176 (0.165, 0.189)	0.154 (0.139, 0.171)

iAUC: integrated AUC; PPV: Positive Predictive Value; NPV: Negative Predictive Value; MCC: Matthew Correlation Coefficient

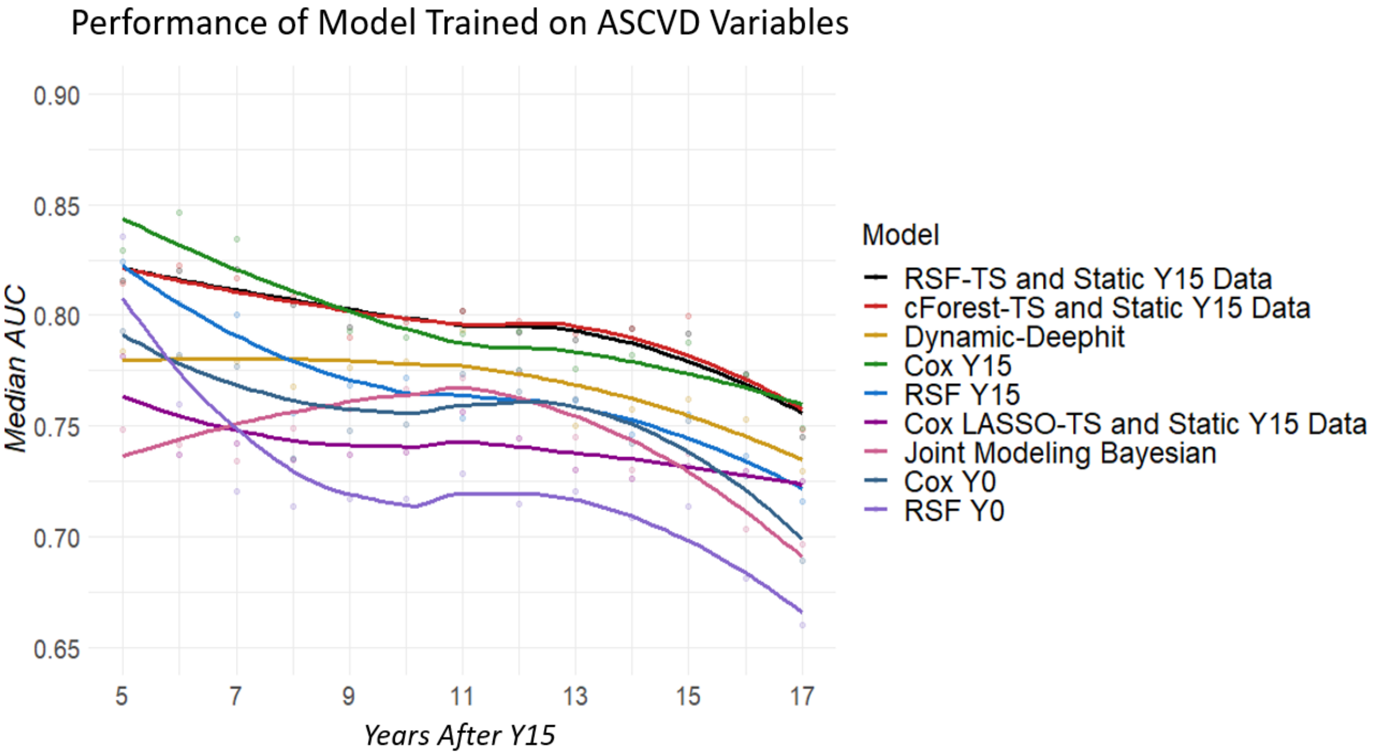

Fig. S5: Model performance over time when limiting the input variables to 9 traditional ASCVD risk factors (Age, gender, race, SBP, cholesterol, HDL, smoking status, diabetes status, and taking high-blood pressure medication status).

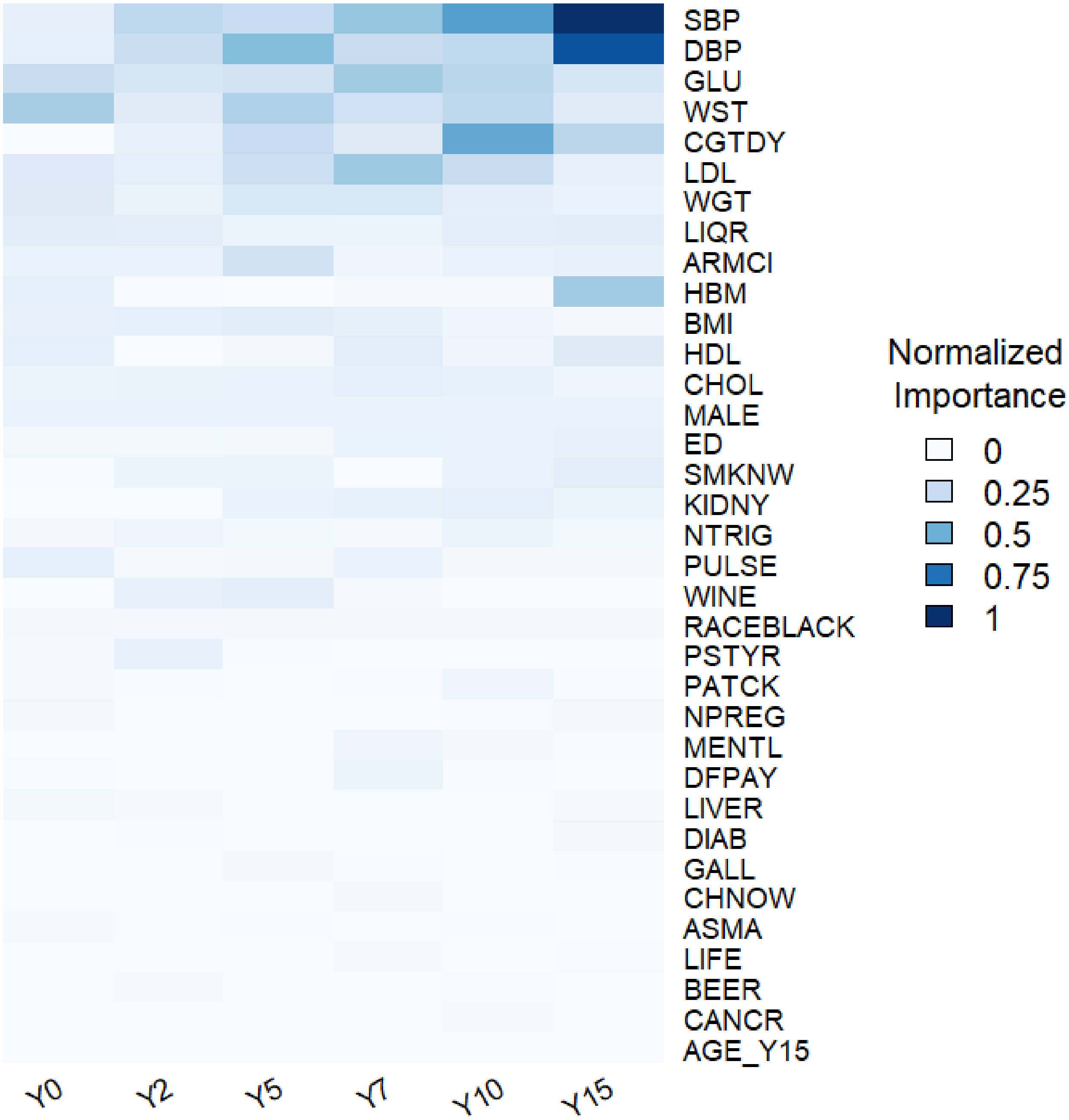

Fig. S6: heatmap showing variable importance for the RSF model trained on concatenated data. Repeated measures (e.g., SBP-Y0, SBP-Y2, SBP-Y5) were treated as independent input variables. RSF-VIMP was used to get the variable importance score for each input variable. All variable importance scores were then normalized between 0-1 and plotted as the z-axis on the heatmap. Variables were ordered along the y-axis based on the averaged importance score across all time points.

Table S3: Race-specific model predictive performance for the top longitudinal modeling strategies (mean and 95% empirical bootstrap interval)

Strategy	Model	iAUC	Post-10 years iAUC	C-index	Last AUC	Brier	Sensitivity	Specificity	PPV	NPV	F1	MCC
Time-series (TS) summary statistics extraction	RSF-TS Black only	0.797 (0.776, 0.816)	0.774 (0.754, 0.791)	0.760 (0.747, 0.77)	0.763 (0.745, 0.779)	0.056 (0.053, 0.058)	0.695 (0.61, 0.792)	0.728 (0.648, 0.803)	0.182 (0.138, 0.218)	0.973 (0.968, 0.977)	0.271 (0.239, 0.3)	0.25 (0.225, 0.274)
	RSF-TS White only	0.790 (0.766, 0.818)	0.779 (0.754, 0.806)	0.765 (0.73, 0.798)	0.74 (0.698, 0.779)	0.031 (0.029, 0.033)	0.747 (0.671, 0.845)	0.644 (0.56, 0.727)	0.086 (0.073, 0.099)	0.985 (0.982, 0.989)	0.152 (0.131, 0.172)	0.166 (0.139, 0.19)
Trajectory clustering	RSF on trajectory clustering data – Black only	0.746 (0.715, 0.777)	0.738 (0.717, 0.761)	0.71 (0.686, 0.733)	0.717 (0.696, 0.735)	0.058 (0.056, 0.059)	0.767 (0.708, 0.841)	0.58 (0.511, 0.648)	0.12 (0.107, 0.132)	0.973 (0.969, 0.978)	0.206 (0.187, 0.223)	0.18 (0.157, 0.2)
	RSF on trajectory clustering data – White only	0.783 (0.748, 0.82)	0.750 (0.698, 0.804)	0.732 (0.692, 0.779)	0.719 (0.676, 0.764)	0.031 (0.03, 0.034)	0.671 (0.585, 0.763)	0.699 (0.639, 0.758)	0.089 (0.073, 0.104)	0.982 (0.978, 0.986)	0.156 (0.131, 0.18)	0.161 (0.127, 0.198)
Data concatenation	RSF on concatenated data – Black only	0.790 (0.756, 0.825)	0.772 (0.745, 0.798)	0.749 (0.727, 0.771)	0.756 (0.732, 0.781)	0.056 (0.055, 0.058)	0.619 (0.548, 0.693)	0.792 (0.738, 0.864)	0.193 (0.165, 0.222)	0.967 (0.963, 0.971)	0.286 (0.258, 0.319)	0.255 (0.224, 0.289)
	RSF on concatenated data – White only	0.792 (0.749, 0.834)	0.784 (0.737, 0.834)	0.764 (0.714, 0.813)	0.749 (0.693, 0.802)	0.031 (0.029, 0.033)	0.727 (0.639, 0.822)	0.713 (0.642, 0.791)	0.105 (0.08, 0.127)	0.985 (0.981, 0.989)	0.18 (0.143, 0.214)	0.197 (0.153, 0.238)
Last observed values	RSF on Y15 data – Black only	0.776 (0.742, 0.813)	0.750 (0.721, 0.778)	0.732 (0.715, 0.75)	0.737 (0.718, 0.757)	0.056 (0.054, 0.058)	0.666 (0.604, 0.731)	0.728 (0.694, 0.765)	0.151 (0.138, 0.165)	0.969 (0.964, 0.973)	0.245 (0.226, 0.264)	0.217 (0.192, 0.241
	RSF on Y15 data – White only	0.783 (0.75, 0.824)	0.752 (0.709, 0.809)	0.739 (0.702, 0.778)	0.729 (0.69, 0.77)	0.031 (0.029, 0.033)	0.628 (0.572, 0.689)	0.76 (0.685, 0.843)	0.121 (0.085, 0.155)	0.981 (0.979, 0.982)	0.194 (0.147, 0.239)	0.195 (0.147, 0.239)

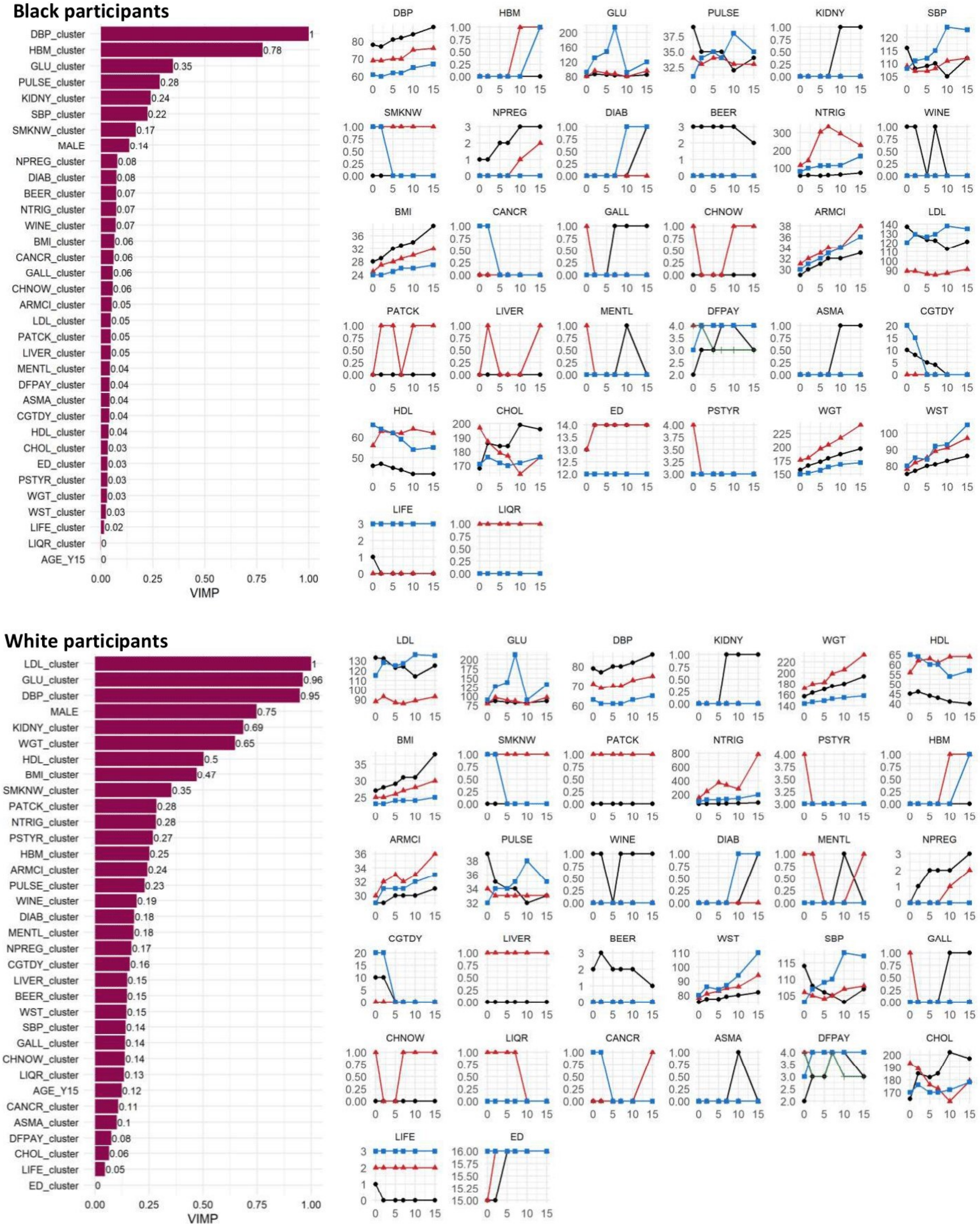

Fig. S7: Explanation for race-specific models of RSF trained on trajectory clustering data. Left panels: normalized median variable importance (VIMP) over 10 folds from permutation for the input variables (trajectory membership and demographic variables) of RSF on trajectory clustering data in Black participants (top) and White participants (bottom). Right panels: cluster profiles for each longitudinal variable, showing the representative (median) trajectory per cluster.
